# Insect-Inspired Self-Motion Estimation with Dense Flow Fields—An Adaptive Matched Filter Approach

**DOI:** 10.1371/journal.pone.0128413

**Published:** 2015-08-26

**Authors:** Simon Strübbe, Wolfgang Stürzl, Martin Egelhaaf

**Affiliations:** 1 Department of Neurobiology and CITEC, Bielefeld University, Bielefeld, Germany; 2 Institute of Robotics and Mechatronics, German Aerospace Center (DLR), Wessling, Germany; Imperial College London, UNITED KINGDOM

## Abstract

The control of self-motion is a basic, but complex task for both technical and biological systems. Various algorithms have been proposed that allow the estimation of self-motion from the optic flow on the eyes. We show that two apparently very different approaches to solve this task, one technically and one biologically inspired, can be transformed into each other under certain conditions. One estimator of self-motion is based on a matched filter approach; it has been developed to describe the function of motion sensitive cells in the fly brain. The other estimator, the Koenderink and van Doorn (KvD) algorithm, was derived analytically with a technical background. If the distances to the objects in the environment can be assumed to be known, the two estimators are linear and equivalent, but are expressed in different mathematical forms. However, for most situations it is unrealistic to assume that the distances are known. Therefore, the depth structure of the environment needs to be determined in parallel to the self-motion parameters and leads to a non-linear problem. It is shown that the standard least mean square approach that is used by the KvD algorithm leads to a biased estimator. We derive a modification of this algorithm in order to remove the bias and demonstrate its improved performance by means of numerical simulations. For self-motion estimation it is beneficial to have a spherical visual field, similar to many flying insects. We show that in this case the representation of the depth structure of the environment derived from the optic flow can be simplified. Based on this result, we develop an adaptive matched filter approach for systems with a nearly spherical visual field. Then only eight parameters about the environment have to be memorized and updated during self-motion.

## 1 Introduction

Knowing one’s self-motion is crucial for navigation, course control and attitude stabilization. Although GPS can provide information about the position and thus about the self-motion of an agent, this information depends on the reliability of the contact to satellites. GPS is not available to animals which have to rely on other means to gain information about their position and self-motion. A direct method to measure self-motion for a walking artificial or biological agent is counting the steps or, in the case of a wheeled vehicle, to monitor the turns of the wheels. In contrast, most flying agents rely on their visual system to solve this task.

The visual system of an artificial or biological agent obtains information about self-motion from pixel shifts in the retinal image over time. These pixel shifts can be described by vectors, the optic flow vectors. The flow vectors depend on both the rotational and translational components of self-motion as well as on the viewing direction. Moreover, for the translational component it also depends on the distance to objects in the environment.

For small translations and rotations, the flow vector for viewing direction $\vec{d_{i}}$ is given by (see [1] for derivation)

$$\begin{array}{ccc} {\vec{p}}_{i}\left(\vec{t},\vec{r},\mu_{i}\right) & = & -\mu_{i}(\vec{t}-(\vec{t}\cdot {\vec{d}}_{i}){\vec{d}}_{i})-\vec{r}\times {\vec{d}}_{i}\\ & = & -\mu_{i}({\vec{d}}_{i}\times \vec{t}\times {\vec{d}}_{i})-\vec{r}\times {\vec{d}}_{i}, \end{array}$$ (1)

where *μ*_*i*_ is the inverse distance (“nearness”) to the object seen in direction $\vec{d_{i}}$, $\vec{t}$ is the translation vector, and $\vec{r}$ is the rotation vector (defining a rotation of angle $r=|\vec{r}|$ around the axis given by $\vec{r}/|\vec{r}|$). According to Eq (1), the flow vector ${\vec{p}}_{i}$ is perpendicular to the corresponding viewing direction $\vec{d_{i}}$. (We use 3d vectors to represent optic flow vectors. Otherwise one would have to define a tangential plane for every viewing direction.)

There are two principally different ways to use optic flow information for self-motion estimation. One way is to identify features in the retinal image at one point in time and find the same features at the next time point in order to compute their displacement. Several technical estimation methods for self-motion are based on these feature correspondences [2, 3]. They rely on a small number of corresponding image points and have to concern about outliers. Such estimation methods are widely used in the technical literature to determine the movement and/or the calibration of a camera [4]. When the self-motion steps are small or the frame rates are high an alternative way to extract self-motion information is possible. Instead of extracting features in the image the local pixel shifts on the retina, called optical flow, produced by the self-motion of the agent is determined through spatiotemporal intensity correspondences in the pattern. This can be done by a gradient-based detector like the Lucas-Kanade detector [5], which compares spatial and temporal derivatives, or by a biologically inspired detector, like the elementary movement detector of the correlation type [6, 7], which uses spatiotemporal auto-correlation signals.

Here we propose a new adaptive approach which combines the advantages of two methods for self-motion estimation based on optical flow, the matched filter approach (MFA) proposed by Franz and Krapp [8] and an algorithm proposed by Koenderink and van Doorn (KvD algorithm) [1]. The MFA estimates self-motion by using linear filters, so called matched filters. Matched filters have the structural form of the pattern they are meant to detect [9] and are the optimal detectors for patterns, which are disturbed by Gaussian errors. In this case the linear filters of the MFA resemble ideal flow fields. Franz and Krapp [8] introduced six filters of this type for the six self-motion components, three for translation and three for rotation. Each of these six filters was tuned to one of the flow fields generated by the six self-motion components, although in general the filters react also to flow generated by the other self-motion components. There is one exception: For a flow field which covers the whole viewing sphere and for isotropic distances, i.e. in the center of a sphere, Borst and Weber [10] showed that model neurons acting as such linear filters are not influenced by other flow fields. To eliminate the influence of other flow fields in the case of an arbitrary field of view Franz et al. [11] introduced a coupling matrix and used its inverse to uncouple the output of the model neurons.

The KvD algorithm is iterative and tries to determine not only the self-motion components but also the distances of the moving agent to objects and surfaces in the environment. These distances influence the translational optic flow and therefore the self-motion estimate. The KvD algorithm starts with a simple distance estimate and determines in the same iteration preliminary self-motion components. In the next iteration these preliminary self-motion components are used as the basis for determining a better distance estimate, which is then used for improving the motion estimate. By the MFA the distances are taken into account statistically and are integrated without further changes in the filters. Dahmen et al. [12] have shown that one iteration step of the KvD algorithm corresponds to the MFA of Franz and Krapp [8] by assuming that some terms in the KvD algorithm are negligibly small. As an important step for the the development of an adaptive approach we show that the MFA with a specific coupling matrix is fully equivalent to one iteration step of the KvD algorithm and not just an approximation. The Gauss-Markov-Theorem [13] gives an explanation of this equivalence. This theorem guarantees the existence of a unique optimal estimator for a linear estimation problem. Both mentioned methods find this optimal solution, although the two approaches seem to be totally different.

The MFA was proposed to explain the motion sensitivity structure of the tangential cells in the fly visual system [8, 14–23]. These cells are directionally selective for motion within their large receptive fields [16, 17, 24–26]. The spatial pattern of their motion sensitivity resembles flow fields on the retina generated by self-motion. Therefore, these cells were proposed to act as matched filters for self-motion estimation and to help the fly to solve visual orientation tasks [8]. However, since the MFA makes a priori assumptions about the 3D structure of the environment, self-motion estimation deteriorates in an environment with variable distance distribution.

It is known that the fly’s nervous system can adapt to sensory inputs [27–32]. With this in mind, we propose a biologically inspired adaptive MFA, which adapts to the depth structure of the environment. This new model avoids the multiple iteration steps used by the KvD algorithm, on the one hand, and the hard-wired distance dependence of the MFA, on the other hand. The adaptive MFA extracts the depth structure from the optic flow field similar to the KvD algorithm. When the distances are not known the self-motion estimation problem becomes non-linear. Although the KvD algorithm is an optimal estimator in the linear case, it is, as we will show, a biased estimator in the non-linear case. The error in the quantities which are estimated does not converge to zero with increasing number of flow vectors. Therefore, we propose a modified version of the KvD algorithm. Numerical simulations indicate that the modified version has no bias.

On the basis of this modified KvD algorithm an adaptive MFA is developed that is inspired by a property of the visual system of insects: Insects have a field of view which nearly covers the whole sphere. It will be discussed that this property is beneficial for self-motion estimation and hence is desirable also for artificial agents which navigate by means of their visual system. The insect or agent should only adapt to the global properties of the depth structure and ignore irrelevant details. To achieve this, the inverse distances are expanded in a complete set of orthonormal functions, the spherical harmonics. It is desirable that the first-orders of this function set contribute most to the solution of the self-motion problem. We show that in the case of spherically distributed flow vectors all orders beyond the second-order of this function set do not contribute to self-motion estimation and can, thus, be neglected without losing information. Hence, if insects or artificial agents adapt to the depth structure they have to be sensitive only to low order depths functions, which are the dipole and quadrupole moments of the depth structure.

## 2 Results

A major objective of this study is to show that two well-established self-motion estimators are mathematically equivalent: The MFA equals one iteration step of the KvD algorithm when the inverse distances to objects in the environment are assumed to be known. To achieve this, we derive the MFA in an alternative way.

We then show that the KvD approach leads to an biased estimator in the general case when distances are unknown and have to be estimated together with the self-motion parameters. We present a modification of the KVD iteration equation that removes the bias and derive an adaptive MFA from this corrected version, which includes a simple but, with respect to self-motion estimation, complete depth model.

Before dealing with these topics, both the basic equations underlying the MFA approach and the KvD algorithm need to be introduced.

### 2.1 The matched filter approach

In the original MFA [8, 11, 33, 34] the depth structure of the environment is not determined from the current flow field but described statistically with a fixed distribution that is assumed to be known. The first statistical parameter considered in [11] is the average inverse distance ${\bar{\mu }}_{i}$, which is measured in every viewing direction over a number of learning flights in different environments. The variability of the distances is given by the covariance matrix *C*_*μ*_. Secondly, the noise in the flow measurement is determined for each viewing direction *n*_*i*_ where a zero mean is assumed. The noise values are combined in the covariance matrix *C*_*n*_. The third statistical parameter is the distribution of the translations *t*. It is assumed that the agent does not translate in every possible direction with the same probability. The corresponding statistical parameter is the covariance matrix *C*_*t*_.

An optic flow vector ${\vec{p}}_{i}$ has only two degrees of freedom because it is the projection of object motion on the retina and thus orthogonal to the corresponding viewing direction ${\vec{d}}_{i}$. To consider only these degrees of freedom Franz et al. [11] introduce a local two-dimensional vector space for each viewing direction ${\vec{d}}_{i}$ which is orthogonal to the direction ${\vec{d}}_{i}$:

$$x_{i}={\vec{p}}_{i}\cdot {\vec{u}}_{i}={\vec{p}}_{i}^{0}\cdot {\vec{u}}_{i}+n_{x,i}$$ (2)

$$y_{i}={\vec{p}}_{i}\cdot {\vec{v}}_{i}={\vec{p}}_{i}^{0}\cdot {\vec{v}}_{i}+n_{y,i}$$ (3)

where $\vec{u}$ and $\vec{v}$ are the basis vectors of the new vector space. The values *x*_*i*_ and *y*_*i*_ represent the two degrees of freedom of ${\vec{p}}_{i}$. The measured vector ${\vec{p}}_{i}$ consists of the true optic flow vector ${\vec{p}}_{i}^{0}$ and an additive noise *n*_*i*_.

In [11] the weights *W* for the matched filters which are multiplied with the optic flow components $\vec{x}$ (where $\vec{x}$ is a 2N dimensional vector containing all flow components *x*_*i*_ and *y*_*i*_, *i* = 1, 2, …, *N*) to estimate the six self-motion components ${\vec{\theta }}^{\text{est}}$,

$${\vec{\theta }}^{\text{est}}=W\cdot \vec{x},$$ (4)

are derived by a least-square principle:

$$e=E\;\left(\|\vec{\theta }-{\vec{\theta }}^{\text{est}}{\|}^{2}\right),$$ (5)

where $\vec{\theta }$ are the true self-motion components. The weight matrix that minimizes the error *e* is:

$$W={\left(F^{T}C^{-1}F\right)}^{-1}\cdot F^{T}\cdot C^{-1}.$$ (6)

The covariance matrix *C* combines the covariance matrices *C*_*μ*_, *C*_*n*_ and *C*_*t*_. The matrix *F* is given by

$$F={\left(\begin{array}{c} -{\bar{\mu }}_{1}{\vec{u}}_{1}\\ {\vec{u}}_{1}\times {\vec{d}}_{1} \end{array}\;\;,\;\begin{array}{c} -{\bar{\mu }}_{1}{\vec{v}}_{1}\\ {\vec{v}}_{1}\times {\vec{d}}_{1} \end{array}\;\;,\;\begin{array}{c} -{\bar{\mu }}_{2}{\vec{u}}_{2}\\ {\vec{u}}_{2}\times {\vec{d}}_{2} \end{array}\;\;,\;\begin{array}{c} -{\bar{\mu }}_{2}{\vec{v}}_{2}\\ {\vec{v}}_{2}\times {\vec{d}}_{2} \end{array}\;\;,\;\ldots \;\right)}^{T},$$ (7)

where ${\bar{\mu }}_{i}$ is the average or expected inverse distance for direction ${\bar{d}}_{i}$. The introduction of the matched filter approach is kept short because an alternative derivation of this approach is introduced in section 2.3, which is more suitable for the comparison with the KvD algorithm.

### 2.2 The Koenderink-van-Doorn (KvD) algorithm

As described by Koenderink and van Doorn [1], a straightforward approach for estimating the self-motion parameters is to find, in accordance with Eq (1), a translation vector $\vec{t}$, a rotation vector $\vec{r}$ and inverse distances {*μ*_*i*_}_*i* = 1, 2, …, *N*_ that minimize the mean squared error between the theoretical optical flow vectors according to Eq (1), ${\left\{{\vec{p}}_{i}(\vec{t},\vec{r},\mu_{i})\right\}}_{i=1,2,\ldots ,N}$ and the measured optical flow vectors ${\left\{{\vec{p}}_{i}\right\}}_{i=1,2,\ldots ,N}$:

$$e\left(\vec{t},\vec{r},\left\{\mu_{i}\right\}\right)=\frac{1}{N}\overset{N}{\underset{i=1}{\sum }}\|{\vec{p}}_{i}\left(\vec{t},\vec{r},\mu_{i}\right)-{\vec{p}}_{i}{\|}^{2}.$$ (8)

$$=\frac{1}{N}\overset{N}{\underset{i=1}{\sum }}\|-\mu_{i}\left(\vec{t}-\left(\vec{t}\cdot {\vec{d}}_{i}\right)\;{\vec{d}}_{i}\right)-\vec{r}\times {\vec{d}}_{i}-{\vec{p}}_{i}{\|}^{2}$$ (9)

Since the optic flow vector (see Eq [1]) depends on the product of $\vec{t}$ and *μ*_*i*_, the same flow vector is obtained by multiplying $\vec{t}$ and dividing *μ*_*i*_ by the same factor. Thus, an additional constraint is imposed to ensure convergence of the minimization procedure. The algorithm described in [1] uses the constraint $\left\|\vec{t}\right\|=1$ and, starting from an initial guess, solves for the motion parameters by iterating the following equations derived from Eq (8) until convergence:

$$\mu_{i}=-\frac{\vec{t}\cdot \left({\vec{p}}_{i}-{\vec{d}}_{i}\times \vec{r}\right)}{1-{\left(\vec{t}\cdot {\vec{d}}_{i}\right)}^{2}},$$ (10)

$$\vec{t}=-\xi \{\langle \mu \vec{p}\rangle +\vec{r}\times \langle \mu \vec{d}\rangle -\langle \mu^{2}\;(\vec{t}\cdot \vec{d})\vec{d}\rangle \},$$ (11)

$$\vec{r}=\langle \vec{p}\times \vec{d}\rangle +\vec{t}\times \langle \mu \vec{d}\rangle +\langle (\vec{r}\cdot \vec{d})\;\vec{d}\rangle .$$ (12)

where *ξ* is a Lagrange multiplier ensuring the constraint $\left\|\vec{t}\right\|=1$. The brackets 〈〉 stand for the average over all viewing directions, i.e. the summation over all directions *i* = 1, 2, …, *N* divided by the number of directions *N*, e.g. $\langle \mu \vec{p}\rangle =\frac{1}{N}\sum_{i=1}^{N}\mu_{i}{\vec{p}}_{i}$.

### 2.3 Alternative derivation and properties of the coupling matrix in the MFA

To compare the two self-motion estimators one needs another mathematical form of the coupling matrix and the filters as given by the MFA (see 2.1). This can be achieved in two different ways. One can either transform the original Eqs (4), (6) and (7) of the MFA or derive the MFA in an alternative manner and show the equivalence to the original MFA afterward. Here, the second way is chosen. The matched filters will be derived according to the theory of optimal filters. The coupling of the estimated self-motion parameters will then be determined by inserting the filters in the flow Eq (1).

The optimal weights in the MFA depend on the statistics of the distances, of the noise and of the preferred translations. Here we assume that nothing is known about these distributions and thus consider the simplest case: The noise values are set to the same value independent of the viewing direction. We assume no preference for specific translation directions. The average inverse distances ${\bar{\mu }}_{i}$ are regarded as known.

The theory of optimal filters states that for a uniform Gaussian noise the best linear filter is a matched filter which has the same form as the pattern it has to detect [9]. Therefore, the templates for estimating translational flow fields have the form of a translational flow field.

$${\vec{T}}_{ia}^{t}=-\mu_{i}\left({\vec{d}}_{i}\times {\vec{e}}_{a}\times {\vec{d}}_{i}\right).$$ (13)

We have three translational templates one for each possible direction of translation represented by the three basis vectors ${\vec{e}}_{a}$ (*a* = 1,2,3).

Similarly, we have three rotational templates

$${\vec{T}}_{ia}^{r}=-{\vec{e}}_{a}\times {\vec{d}}_{i}.$$ (14)

The three translational and three rotational templates ${\vec{T}}_{a}^{t}$ and ${\vec{T}}_{a}^{r}$ will be called “standard templates”.

The scalar product $\langle \vec{T\cdot }\vec{p}\rangle$ of a flow field $\vec{p}$ and a template $\vec{T}$, where the brackets stand for the mean over all viewing directions, can be interpreted as the output of a specific model $\text{neuron}a=\langle \vec{T}\cdot \vec{p}\rangle$. In general, the model neurons do not only react to the flow fields they are tuned to, but also to other flow fields. To solve this problem Franz et al. [11] introduced a matrix (*F* ^*T*^ *C* ^−1^ *F*)^−1^ (see Eq (6)) which compensates for the coupling to other flow fields. The coupling between the self-motion estimates and therefore the coupling matrix are determined be inserting the templates ${\vec{T}}_{a}^{t}$ and ${\vec{T}}_{a}^{r}$ in the flow Eq (1). For this, the translation $\vec{t}$ and the rotation $\vec{r}$ must be separated into their components, $\vec{t}=t_{1}{\vec{e}}_{1}+t_{2}{\vec{e}}_{2}+t_{3}{\vec{e}}_{3}$ and $\vec{r}=r_{1}{\vec{e}}_{1}+r_{2}{\vec{e}}_{2}+r_{3}{\vec{e}}_{3}$, with the six self-motion parameters *t*_1_, *t*_2_, *t*_3_ and *r*_1_, *r*_2_, *r*_3_.

Since the cross product is linear $\left(\alpha \;\vec{a}+\beta \;\vec{b}\right)\times \vec{c}=\alpha \left(\vec{a}\times \vec{c}\right)+\beta \left(\vec{b}\times \vec{c}\right)$, the overall flow field is the sum of the six standard templates ${\vec{T}}_{A}$ (*A* = 1, 2, …, 6) weighted by the six self-motion components *θ*_*A*_:

$$\begin{array}{ccc} {\vec{p}}_{i} & = & t_{1}{\vec{T}}_{i1}^{t}+t_{2}{\vec{T}}_{i2}^{t}+t_{3}{\vec{T}}_{i3}^{t}+r_{1}{\vec{T}}_{i1}^{r}+r_{2}{\vec{T}}_{i2}^{r}+r_{3}{\vec{T}}_{i3}^{r}\;,\\ & = & \theta_{1}{\vec{T}}_{i1}+\theta_{2}{\vec{T}}_{i2}+\theta_{3}{\vec{T}}_{i3}+\theta_{4}{\vec{T}}_{i4}+\theta_{5}{\vec{T}}_{i5}+\theta_{6}{\vec{T}}_{i6}. \end{array}$$ (15)

Following our notation, the response of model neuron *a*_*A*_ with corresponding template ${\vec{T}}_{A}$ to the flow field $\vec{p}$ (Eq (15)) is

$$a_{A}=\langle {\vec{T}}_{A}\cdot \vec{p}\rangle =\langle {\vec{T}}_{A}\cdot \sum_{B}{\vec{T}}_{B}\theta_{B}\rangle =\sum \langle {\vec{T}}_{A}\cdot {\vec{T}}_{B}\rangle \;\theta_{B}=\sum_{B}{\hat{M}}_{AB}\theta_{B}.$$ (16)

Combining the responses of all six model neurons in one equation gives

$$\vec{a}=\hat{M}\cdot \vec{\theta },$$ (17)

where the vectors $\vec{a}$ and $\vec{\theta }$ are considered as six dimensional vectors. Each entry of the 6 × 6 dimensional matrix $\hat{M}$, the coupling matrix, can be seen as the generalized scalar product of two of the six standard templates:

$${\hat{M}}_{AB}=\langle {\vec{T}}_{A}\cdot {\vec{T}}_{B}\rangle$$ (18)

We can also write the coupling matrix as

$$\begin{array}{ccc} M & = & (\begin{array}{cc} M^{tt} & M^{tr}\\ M^{rt} & M^{rr} \end{array}), \end{array}$$ (19)

where the indices *t* and *r* of the 3 × 3 sub-matrices indicate which templates are multiplied.

Using the inverse of the coupling matrix we can estimate the motion parameters $\vec{\theta }$ from the responses of the model neurons,

$${\vec{\theta }}^{\text{est}}={\hat{M}}^{-1}\vec{a}={\hat{M}}^{-1}\;(\begin{array}{c} \langle {\vec{T}}_{1}^{t}\cdot \vec{p}\rangle \\ \langle {\vec{T}}_{2}^{t}\cdot \vec{p}\rangle \\ \langle {\vec{T}}_{3}^{t}\cdot \vec{p}\rangle \\ \langle {\vec{T}}_{1}^{r}\cdot \vec{p}\rangle \\ \langle {\vec{T}}_{2}^{r}\cdot \vec{p}\rangle \\ \langle {\vec{T}}_{3}^{r}\cdot \vec{p}\rangle \end{array}).$$ (20)

The product $\langle \vec{T}\cdot \vec{p}\rangle$ and the multiplication with ${\hat{M}}^{-1}$ are linear transformations of the optical flow. One can therefore define new templates *T*′ which include the linear transformation given by the matrix ${\hat{M}}^{-1}$.

Dahmen et al. [12] tested previously the self-motion estimation performance for different fields of view. The self-motion estimation performance even for error prone flow vectors is high, if the flow fields corresponding to different self-motion components differ essentially over the field of view. For a restricted field of view, for example a small region in front of the agent, upward translation cannot be distinguished from a pitch rotation of the agent. In this case the coupling matrix with constant distances becomes nearly singular and cannot be properly inverted.

In section 5.4 of the appendix it is shown by means of a coordinate transformation of two dimensional flow vectors (in tangent planes) into three dimensional ones (on the sphere) that Eq (20) is equivalent to Eq (4) with the weights given by Eq (6).

#### 2.3.1 Properties of the coupling matrix

The equivalence between the MFA and one iteration of the KvD algorithm is shown in three steps. The first step was the alternative derivation of the MFA described above. The following second step is a simplification of the four sub-matrices of the coupling matrix.

Three cases have to be considered when calculating the entries of the matrix: the scalar product between two translational templates, the scalar product between two rotational templates and the scalar product between a translational and a rotational template.

The scalar product between two translational templates leads to the following expression:

$$\begin{array}{lll} {\left(M^{tt}\right)}_{ab} & = & \langle {\vec{T}}_{a}^{t}\cdot {\vec{T}}_{b}^{t}\rangle \\ & = & \langle \mu^{2}\rangle {\vec{e}}_{a}\cdot {\vec{e}}_{b}-\langle \mu^{2}\left({\vec{e}}_{a}\cdot \vec{d}\right)\cdot \left({\vec{e}}_{b}\cdot \vec{d}\right)\rangle . \end{array}$$ (21)

The scalar product between two rotational templates results in

$$\begin{array}{lll} {\left(M^{rr}\right)}_{ab} & = & \langle {\vec{T}}_{a}^{r}\cdot {\vec{T}}_{b}^{r}\rangle \\ & = & {\vec{e}}_{a}\cdot {\vec{e}}_{b}-\langle \left({\vec{e}}_{a}\cdot \vec{d}\right)\cdot \left({\vec{e}}_{b}\cdot \vec{d}\right)\rangle . \end{array}$$ (22)

Finally, the scalar product between a translational and a rotational template leads to

$$\begin{array}{ccc} {\left(M^{tr}\right)}_{ab} & = & {\left(-M^{rt}\right)}_{ab}=\langle {\vec{T}}_{a}^{t}\;\cdot \;{\vec{T}}_{b}^{r}\rangle \\ & = & \left({\vec{e}}_{b}\times {\vec{e}}_{a}\right)\cdot \langle \mu \vec{d}\rangle . \end{array}$$ (23)

Eq (21) informs us that the estimates of the three translation parameters *t*_1_, *t*_2_ and *t*_3_ are coupled unless the term $\langle \left({\vec{e}}_{a}\cdot \vec{d}\right)\cdot \left({\vec{e}}_{b}\cdot \vec{d}\right)\rangle$ in *M* ^*tt*^ is proportional to the identity matrix. The same holds for the coupling between the three rotation parameters *r*_1_, *r*_2_ and *r*_3_ described by *M* ^*rr*^, see Eq (22). Similarly, the term $\langle \mu \vec{d}\rangle$ in *M* ^*tr*^, Eq (23), must be zero for the translation and rotation estimates to be uncoupled.

#### 2.3.2 The case of constant distances and a spherical field of view

Borst and Weber [10] showed that for viewing directions homogeneously covering the whole sphere, and for identical distances (*μ*_*i*_ = constant for all *i*) the model neurons respond only to the components of the flow field they are tuned to. This result can be easily verified within the conceptual framework provided here by replacing the sums in Eqs (21), (22) and (23) with integrals over the unit sphere and by introducing spherical coordinates. The direction vectors ${\vec{d}}_{i}$ are then replaced by the vectors ${\vec{d}}_{\vartheta \varphi }$ that depend on the elevation angle *ϑ* and azimuth angle *φ*. In appendix 5.3 it is shown that the direction vectors ${\vec{d}}_{\vartheta \varphi }$ in the spherical coordinate system have the same form as the three real-valued dipole functions of the spherical harmonics. Due to the orthogonality of the spherical harmonics the integral $\int \left({\vec{e}}_{a}\cdot {\vec{d}}_{\vartheta \varphi }\right)\cdot \left({\vec{e}}_{b}\cdot {\vec{d}}_{\vartheta \varphi }\right)$ sin *ϑdϑdφ* becomes zero in the case of spherically distributed flow vectors, if ${\vec{e}}_{a}$ and ${\vec{e}}_{b}$ denote different basis vectors. The scalar product between a translational and a rotational template Eq (23) leads to the integral $\int \left({\vec{e}}_{b}\times {\vec{e}}_{a}\right)\cdot {\vec{d}}_{\vartheta \varphi }\;\text{sin}\;\vartheta d\vartheta d\varphi$ which can be regarded as the product between a first-order dipole function and the zeroth order spherical harmonic function (which is a constant). Due to the orthogonality of the spherical harmonics this integral is zero.

### 2.4 The relationship between the MFA and the KvD algorithm

In a final step we will show that the MFA with the coupling matrix is equivalent to one iteration of the KvD algorithm [1] for known distances. Hence, the two approaches do not represent principally different methods, but rather one method with two different ways of dealing with the depth structure. An additional way to take the depth structure into account is given in section 2.6 where an adaptive MFA is proposed.

Dahmen et al. [12] have already demonstrated that a MFA can be derived from the KvD algorithm, if certain terms are small and can be neglected. We will show that these terms are identical to entries of the coupling matrix, which was derived in the previous chapter.

#### The equivalence of the MFA and one iteration step of the KvD algorithm

Not only in the MFA, but also in the KvD approach coupling terms can be identified. In the translation Eq (11), the second term on the right side comprises the rotation. The rotation Eq (12) in turn contains a translational term. These terms were called “apparent rotation” and “apparent translation” by Dahmen et al. [12], and they couple translation and rotation. The third terms in both equations couple different components of the translation or the rotation whenever $\langle \mu^{2}\vec{d}⊗\vec{d}\rangle$ in $\langle \mu^{2}\left(\vec{t}\cdot \vec{d}\right)\;\vec{d}\rangle =\langle \mu^{2}\vec{d}⊗\vec{d}\rangle \cdot \vec{t}$ or $\langle \vec{d}⊗\vec{d}\rangle$ in $\langle \left(\vec{r}\cdot \vec{d}\right)\;\vec{d}\rangle =\langle \vec{d}⊗\vec{d}\rangle \cdot \vec{r}$ contain off-diagonal components. The following derivation will show that these coupling terms are equivalent to the terms of the coupling matrix in the MFA.

The derivation starts with the MFA including the coupling matrix. From Eq (17) one obtains together with the equations for the coupling matrix Eqs (21), (22) and (23):

$$\begin{array}{c} \vec{a}\;=\;\hat{M}\cdot \vec{\theta }=(\begin{array}{cc} M^{tt} & M^{tr}\\ M^{rt} & M^{rr} \end{array})\cdot (\begin{array}{c} \vec{t}\\ \vec{r} \end{array})\;=\;(\begin{array}{c} \langle {\vec{T}}_{1}^{t}\cdot \vec{p}\rangle \\ \langle {\vec{T}}_{2}^{t}\cdot \vec{p}\rangle \\ \langle {\vec{T}}_{3}^{t}\cdot \vec{p}\rangle \\ \langle {\vec{T}}_{1}^{r}\cdot \vec{p}\rangle \\ \langle {\vec{T}}_{2}^{r}\cdot \vec{p}\rangle \\ \langle {\vec{T}}_{3}^{r}\cdot \vec{p}\rangle \end{array}) \end{array}$$ (24)

$$\begin{array}{c} {\left(M^{tt}\right)}_{ab}=\langle \mu^{2}\rangle {\vec{e}}_{a}\cdot {\vec{e}}_{b}-\langle \mu^{2}\;\left({\vec{e}}_{a}\cdot \vec{d}\right)\cdot \left({\vec{e}}_{b}\cdot \vec{d}\right)\rangle \end{array}$$ (25)

$${\left(M^{tr}\right)}_{ab}=\langle \mu \;\left({\vec{e}}_{a}\times {\vec{e}}_{b}\right)\;\cdot \;\vec{d}\rangle$$ (26)

$${\left(M^{rt}\right)}_{ab}=-\langle \mu \;\left({\vec{e}}_{a}\times {\vec{e}}_{b}\right)\;\cdot \;\vec{d}\rangle$$ (27)

$${\left(M^{rr}\right)}_{ab}={\vec{e}}_{a}\cdot {\vec{e}}_{b}-\langle \left({\vec{e}}_{a}\cdot \vec{d}\right)\cdot \left({\vec{e}}_{b}\cdot \vec{d}\right)\rangle$$ (28)

On the left side of Eq (24), the vectors $\vec{t}$ and $\vec{r}$ are multiplied with the entries of the coupling matrix. On the right side the optical flow vectors $\vec{p}$ are multiplied with the templates. After some algebraic simplifications, which are given in appendix 5.1, and a rearrangement of the terms one obtains the known equations for translation Eq (11) and rotation Eq (12) of the KvD algorithm:

$$\vec{t}=-\frac{1}{\langle \mu^{2}\rangle }\;\{\langle \mu \vec{p}\rangle +\vec{r}\times \langle \mu \vec{d}\rangle -\langle \mu^{2}\left(\vec{t}\cdot \vec{d}\right)\;\vec{d}\rangle \},$$

$$\vec{r}=\langle \vec{p}\times \vec{d}\rangle +\vec{t}\times \langle \mu \vec{d}\rangle +\langle (\vec{r}\cdot \vec{d})\;\vec{d}\rangle ,$$

where $\frac{1}{\langle \mu^{2}\rangle }$ represents the Lagrange multiplier *ξ*.

Hence, the MFA and the KvD algorithm are identical for one iteration step.

### 2.5 The bias of the KvD algorithm

The equivalence of the KvD algorithm and the MFA for known distances follows directly from the Gauss-Markov theorem [13], which states that an ordinary least-squares estimator is the best unbiased estimator for an estimation problem which is linear and has uncorrelated errors with equal variances. Both methods start with such a least-squares approach. The MFA minimizes the quadratic error between the six true self-motion values and the estimated values as can be seen in Eq (5), whereas the KvD algorithm minimizes the quadratic error between the measured optic flow and the theoretical optic flow as can be seen in Eq (8). For known distances, the optic flow and the self-motion values are connected through a linear transformation. Thus, the two least-square approaches lead to the same self-motion estimator. This estimator is the unique optimal estimator as stated by the Gauss-Markov theorem.

The situation is different if the distances are not known and have to be estimated by the estimator together with the self-motion values. Then the problem is no longer linear and the Gauss-Markov theorem does not hold. Nonetheless, it seems likely that a least-square approach like in Eq (8) leads to an optimal estimator in the sense that the error in the estimated self-motion components approaches zero with increasing number of measured optical flow values. However, as will be shown in this section, the KvD algorithm in general does not converge to the true self-motion values for an infinite number of flow vectors. It is still an open question from which minimization principle an optimal estimator can be derived. The increasing number of flow vectors raises the problem that for each flow vector which gives two additional error-prone values one additional value has to be estimated: the inverse distance in the respective viewing direction. Although the number of measured values increases towards infinity, the ratio between the number of estimated and measured values does not decrease to zero. Hence, even for an infinite number of flow vectors the estimated inverse distances are still afflicted with errors. However, it should still be possible to correctly estimate the fixed number of self-motion values for an infinite number of flow vectors. In section 2.5.2 a modified KvD algorithm will be derived. The modification is tested numerically under two conditions where the original KvD algorithm turns out to be biased (section 2.5.3).

#### 2.5.1 The non-vanishing error term

The KvD algorithm is an unbiased estimator only under certain conditions. To show this, the propagation of the error in the flow vectors ${\vec{p}}_{i}$ over the iterations will be analyzed. We model the measured flow vectors ${\vec{p}}_{i}$ as the sum of the true vector ${\vec{p}}_{i}^{0}$ and a random error vector $\Delta {\vec{p}}_{i}$, ${\vec{p}}_{i}={\vec{p}}_{i}^{0}+\Delta {\vec{p}}_{i}$. Similar to vector ${\vec{p}}_{i}$, the vectors ${\vec{p}}_{i}^{0}$ and $\Delta {\vec{p}}_{i}$ have only two degrees of freedom. It will be assumed that the random vectors $\Delta {\vec{p}}_{i}$ are unbiased, i.e. the expectation values for all directions *i* are zero, $E\;\left(\Delta {\vec{p}}_{i}\right)=0$.

Two special conditions will be considered in the following:

1. The viewing directions $\vec{d_{i}}$ are equally distributed over the sphere.
2. The random vectors $\Delta {\vec{p}}_{i}$ are uncorrelated and their variances constant, independent of the directions *i*, $\text{var\textbackslash{}!}\left(\Delta {\vec{p}}_{i}\right)=E\;\left(\Delta {\vec{p}}_{i}\cdot \Delta {\vec{p}}_{i}\right)=\text{constant}$.

The Gauss-Markov theorem assumes condition (2) to be fulfilled. We will also consider deviations from this condition, because the KvD algorithm and its modified version that will be derived in section 2.5.2 behave differently then. Condition (2) is violated if, for example, the error of the optic flow measurement depends on the length of the measured optic flow vectors.

In subsection 5.2 of the appendix it is shown that, in general, the translation estimated by the KvD algorithm contains errors that do not vanish even for an increasing number of flow vectors. There are two error terms which are additive to the real translation ${\vec{t}}^{0}$.

$$\vec{t}={\vec{t}}^{0}+\Delta \vec{t}={\vec{t}}^{0}+a+b$$

$$a\propto {\langle \frac{\Delta \vec{p}⊗\Delta \vec{p}}{1-{\left({\vec{t}}^{0}\cdot \vec{d}\right)}^{2}}\rangle }_{\infty }\cdot {\vec{t}}^{0}$$

$$b\propto {\langle \frac{{\vec{t}}^{0}\cdot (\Delta \vec{p}⊗\Delta \vec{p})\cdot {\vec{t}}^{0}}{{\left(1-{\left({\vec{t}}^{0}\cdot \vec{d}\right)}^{2}\right)}^{2}}(\vec{d}⊗\vec{d})\rangle }_{\infty }\cdot {\vec{t}}^{0}$$

The index ∞ of the brackets 〈〉 stands for the limit of an infinite number of flow vectors. The discrete direction vectors ${\vec{d}}_{i}$ can be exchanged by their continuous counterparts ${\vec{d}}_{\vartheta \varphi }$, and the sum over *i* can be replaced by an integral over the field of view.

The iteration equation for $\vec{r}$, Eq (12), does not contain terms that lead to a bias in the estimated motion values. However, the estimated rotation will be affected indirectly by errors in the estimated translation.

First the integrals containing the denominators $D_{1}=1-{\left({\vec{t}}^{0}\cdot \vec{d}\right)}^{2}$ and $D_{2}={\left(1-{\left({\vec{t}}^{0}\cdot \vec{d}\right)}^{2}\right)}^{2}$ are analyzed, disregarding the numerators. The integrals over $\frac{1}{D_{1}}$ and $\frac{1}{D_{2}}$ are zero only if the first condition of equally distributed flow vectors is fulfilled. To avoid the singularity for $\vec{d}={\vec{t}}^{0}$ a small constant *ɛ* was added to the denominators.

If conditions (1) and (2) are fulfilled, the terms *a* and *b* are zero and the KvD algorithm is an unbiased estimator.

If condition (1) holds but condition (2) does not as, for instance, in the case of realistic EMDs or gradient-based detectors, we have to integrate over a direction dependent function resulting from the direction dependent flow errors $\Delta \vec{p}$. Hence the terms *a* and *b* converge to finite values.

The error terms *a* and *b* do not play a role if they are proportional to the identity matrix, because of the rescaling of the translation vector, which ensures $\left\|\vec{t}\right\|=1$. The matrices $E_{1}=\langle \Delta \vec{p}⊗\Delta \vec{p}\rangle$ and $E_{2}=\langle \left(\Delta \vec{p}⊗\Delta \vec{p}\right)\cdot (\vec{d}⊗\vec{d})\rangle$ are proportional to the unit matrix, if and only if both conditions (1) and (2) are satisfied. This can be shown by taking into account the symmetry of viewing directions and the constant variances of the flow errors.

Most interestingly, if condition (2) is fulfilled (a pre-condition of the Gauss-Markov theorem) but condition (1) is not, the terms *a* and *b* converge to finite values. In this case the integrals over the denominators *D*_1_, *D*_2_ and the integrals over the numerators *E*_1_, *E*_2_ have finite values. This means that the ordinary least-squares approach from Eq (8) leads to a biased self-motion estimator.

#### 2.5.2 Modification of the KvD iteration equations

To improve the KvD algorithm a modified version of the iteration Eq (11) will be derived. The flow Eq (1) can be transformed:

$${\vec{p}}_{i}=-\mu_{i}\;(\vec{t}-(\vec{t}\cdot {\vec{d}}_{i})\;{\vec{d}}_{i})-\vec{r}\times {\vec{d}}_{i}$$ (29)

$$\mu_{i}\vec{t}=-{\vec{p}}_{i}-\vec{r}\times {\vec{d}}_{i}+\mu_{i}\;(\vec{t}\cdot {\vec{d}}_{i})\;{\vec{d}}_{i}\;.$$ (30)

By taking the average and solving for $\vec{t}$ we obtain

$$\langle \mu \vec{t}\rangle =-\langle \vec{p}\rangle -\langle \vec{r}\times \vec{d}\rangle +\langle \mu (\vec{t}\cdot \vec{d})\;\vec{d}\rangle ,$$

$$\vec{t}={\langle \mu \rangle }^{-1}\;\{-\langle \vec{p}\rangle -\langle \vec{r}\times \vec{d}\rangle +\langle \mu \left(\vec{t}\cdot \vec{d}\right)\;\vec{d}\rangle \},$$

$$\vec{t}=-\xi \{\langle \vec{p}\rangle +\vec{r}\times \langle \vec{d}\rangle -\langle \mu \;(\vec{t}\cdot \vec{d})\;\vec{d}\rangle \},$$ (31)

where *ξ* ensures that $\vec{t}$ is normalized. Compared with the original equation for the translation Eq (11) the additional factor *μ*_*i*_ is absent. Nonetheless, the above equation for the translation depends still on the distances. An analog derivation leads to the same iteration equation for the rotation as in the original KvD algorithm.

If the flow vectors have no errors the modified version converges, as does the original algorithm, to the true self-motion parameters. In contrast to the result for the original KvD algorithm in appendix 5.2, the iteration equations of the modified version do not contain $\mu \vec{p}$ or *μ* ^2^ and the true self-motion values are fix points of the iteration (only when the true values are fix points in the iteration the algorithm can converge to these values).

#### 2.5.3 Numerical tests of the original and modified KvD algorithm

**Fig 1:**
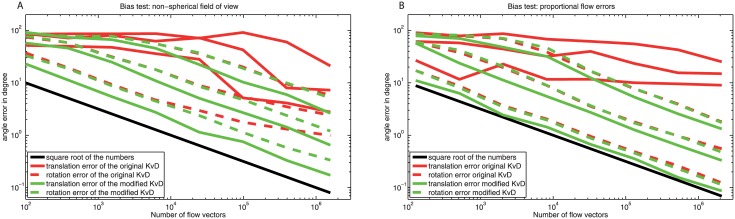
The averaged angle error, $\text{arccos}\left(\frac{{\vec{\theta }}_{N}^{\text{est}}\cdot \vec{\theta }}{|{\vec{\theta }}_{N}^{\text{est}}|\cdot |\vec{\theta }|}\right)$, between the estimation ${\vec{\theta }}_{N}^{\text{est}}$, which depends on the number of flow vectors *N*, and the true values $\vec{\theta }$, is shown for translation (solid lines) and rotation (dashed lines). Red curves show the errors for original KvD algorithm, green curves for the modified KvD algorithm. The errors are averaged over 40 trials (see methods 4.3). For each trial the true self-motion parameters are chosen randomly, equally distributed over the sphere, in such a way, that the resulting translational flow equals the resulting rotational flow in magnitude. The distances, also determined randomly, lie with equal probability between one and three in arbitrary units. The results for three different variances ${\left(△\vec{p}\right)}^{2}$ are shown (from bottom to top): 1, 3, and 9 times the flow vector length, where the factor is interpreted differently in the two graphs. **A)** Results for non-equally distributed flow vectors with equal variance of the flow vector errors $△{\vec{p}}_{i}$ (the variance is matched to the mean flow vector). **B)** Results for equally distributed flow vectors, where the variance of the errors $△{\vec{p}}_{i}$ depends linearly on the length of the ${\vec{p}}_{i}$ (the variance is matched to the local flow vector).

In Fig 1 the bias of the KvD algorithm is shown numerically (see section ‘Material and Methods’, 4.3 for a detailed description of the numerical test). The left part of the figure shows simulation results for flow vectors with added errors of equal variance. The field of view given by the viewing directions ${\vec{d}}_{i}$ is non-equally distributed: The flow vectors are equally distributed except for two regions of the sphere which do not contain any flow vectors. The two regions are quarters of the half-sphere which lie opposite to each other in the upper half-sphere. Thus, the simulation result provides an example where condition (2) of section 2.5.1 is fulfilled but condition (1) is not. The translation error of the original KvD algorithm is significantly larger and increasingly deviates from that of the modified version with increasing number of flow vectors. Due to the coupling of the iteration equations, the error of the rotation, in case of the original KvD algorithm, is affected by the translation error and, thus, also deviates from that of the modified version.

The right part of the figure shows results where the standard deviation of $△{\vec{p}}_{i}$ is proportional to the length of the local flow vector ${\vec{p}}_{i}$. This time, the viewing directions cover the whole sphere homogeneously and, thus, condition (1) of section 2.5.1 holds while condition (2) does not. Again, the original KvD algorithm is biased (see also section 2.5.1). However, the error of the rotation in the original KvD algorithm is not influenced by the translation error as a consequence of the spherically distributed viewing directions.

The error of the modified KvD algorithm is in both analyzed cases inversely proportional to the square root of the number of flow vectors (see black line in Fig 1). The modified KvD algorithm shows therefore an error behavior as is characteristic of an unbiased linear estimator.

### 2.6 An adaptive MFA

The approach of Franz and Krapp [8] is based on the assumption that the statistics of the depth structure of the environment is fixed as well as the preferred translation directions of the agent are known. For the simplest statistical model, which assumes that the distance variability, the noise in the flow measurements and the preferred translation of the agent are independent from the viewing direction, the dedicated covariance matrices are proportional to the identity matrix. Most important, one has to specify the depth structure of the environment by defining the average inverse distances 〈*μ*〉_*i*_ or, as in [8], the average distances 〈*D*〉_*i*_. Franz and Krapp [8] modeled the distances 〈*D*〉_*i*_ by

$${\langle D\rangle }_{i}=\{\begin{array}{cc} D_{0} & \varepsilon_{i}\geq 0\;,\\ \frac{\beta D_{0}}{\sqrt{1+(\beta^{2}-1)\cos^{2}(\varepsilon_{i})}} & \varepsilon_{i}<0, \end{array}$$ (32)

where *D*_0_ denotes a typical distance in the upper hemisphere, *ɛ*_*i*_ the elevation angle of viewing direction *i* and $\beta =\frac{h}{D_{0}}<1$ the ratio of the average flight altitude *h* and *D*_0_. This model takes into account that the distances are usually smaller for viewing directions below the horizon.

It is obvious that the performance of self-motion estimation is poor when the fixed depth model Eq (39) is not a good description of the depth structure at the current position of the agent. Therefore, we propose an adaptive MFA, which allows the agent to adapt its depth model to the current environment. This is only possible, if we can assume that the depth structure properties of the environment do not change abruptly from one time step to the next. A time constant describes the intervals in which the depth model will be updated. Between updates the depth model remains fixed and has to be memorized by the agent. The depth information is obtained from the optic flow, as well as the self-motion values. Since the original KvD algorithm is biased in this case, we use our modified KvD version as the starting point for deriving the adaptive model.

As will be shown in the following, it is not necessary to represent the full depth information, because it is sufficient to memorize just eight distance dependent parameters. Three parameters are contained in the distance-dependent term of the rotation Eq (12), $\vec{t}\times \langle \mu \vec{d}\rangle$ or (*t*_1_, *t*_2_, *t*_3_)^*T*^ × (*μ* sin (*ϑ*) cos (*φ*), *μ* sin (*ϑ*) sin (*φ*), *μ* cos (*ϑ*))^*T*^. In the translation equation of the modified KvD algorithm Eq (31) only $\langle \mu \vec{d}⊗\vec{d}\rangle \cdot \vec{t}$ depends on *μ*. As the matrix $\langle \mu \vec{d}⊗\vec{d}\rangle$ is symmetric it contains only six different *μ*-dependent elements.

The first three orders of spherical harmonic functions, the zeroth, first and second order, comprise nine parameters, but only eight parameters can be determined from the optic flow. The zeroth order remains undefined, because one cannot distinguish between the situation, where all objects have half the distance to the agent, and the situation, where the agent translates with double speed: The optical flow will be the same. Hence, from optic flow fields only the direction of the translation can be identified.

Between updates of the depth model, rotations, in particular, impair its validity, but can be easily compensated by counter-rotating the depth model. This is achieved by multiplying the depth dependent coupling matrix with a rotation matrix obtained from the rotation parameters of the current self-motion estimate.

The adaptive model will be derived first for the general case with an arbitrary field of view. Then a more biologically plausible adaptive model with a spherical field of view will be presented. A spherical field of view facilitates an intuitive interpretation of the depth model. In this specific case, the nine depth-dependent parameters are exactly the coefficients of the first three orders of the spherical harmonics expansion, i.e the dipole and quadrupole moments of the depth structure *μ*_*ϑφ*_.

#### 2.6.1 Motivation for an adaptive MFA

**Fig 2:**
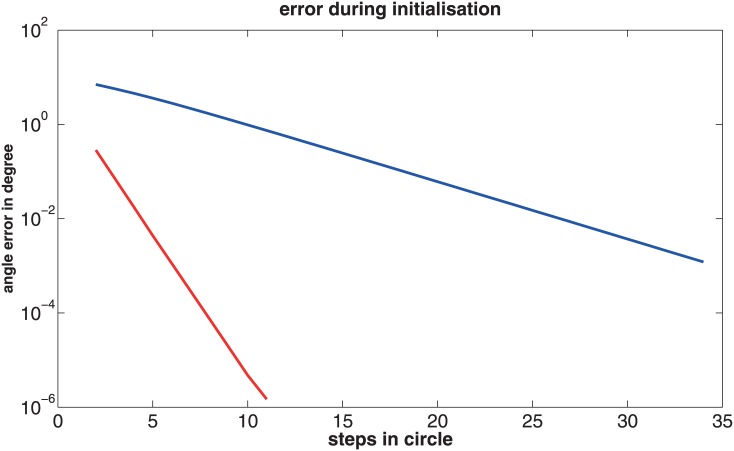
An agent flies inside the sphere on a circular trajectory (see Fig 3A). The center of the circle does not coincide with the center of the sphere to avoid symmetries in the depth model. The correct depth model is constant in this configuration, only the initialization of the depth model is tested. The y-axis shows the angle error $\text{arccos}\left(\frac{{\vec{\theta }}^{\text{est}}\cdot \vec{\theta }}{|{\vec{\theta }}^{\text{est}}|\cdot |\vec{\theta }|}\right)$, between the estimated self-motion axis of ${\vec{\theta }}^{\text{est}}$ and the axis of the true self-motion values $\vec{\theta }$. The depth model is initialized with constant distances. With every update step of the depth model the error decreases exponentially for the translation (blue) as well as for the rotation (red).

**Fig 3:**
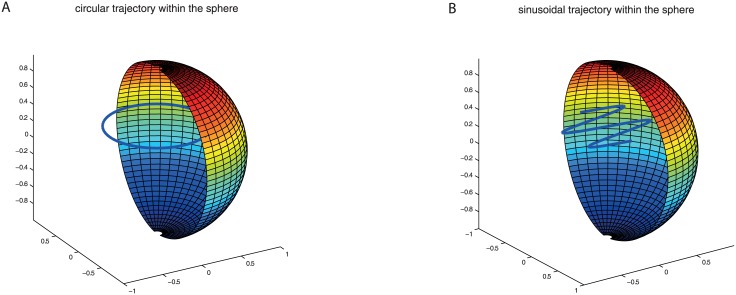
**A)** shows a circular trajectory to analyze the initialization phase of the adaptive MFA. The height of the trajectory lies above the middle point of the sphere to avoid trivial depth models. Due to symmetry the depth model for this configuration is the same at every trajectory point. **B)** shows a sinusoidal trajectory. It is used to analyze the self-motion estimation error during adaptation. Again the height of the trajectory is lifted up against the middle point of the sphere to make the depth model more complex in relation to an agent, which flies along the trajectory.

In Fig 2 the initialization phase of the adaptive model is shown. The agent flies inside a sphere in such a way, that the depth model is the same for every trajectory step (see Figs 2 and 3B and methods 4.4 for details). The error in the estimated self-motion parameters decreases exponentially. Hence, the error is corrected to a large extent in the first few iteration steps.

In natural environments one can detect subspaces where the distances to a moving agent do not change over a certain time [35]. Hence, one can assume that the overall depth model changes only slightly from one step to the next in a given environment and can expect good self-motion estimates even for only a single iteration step of the KvD algorithm based on the old depth model. Furthermore, the depth model can be used even for a longer time interval. In this case the depth model is not updated instantaneously after receiving new optic flow information, but less frequently after several optic flow processing steps. Because the self-motion parameters and the depth model are formulated in the body coordinate system of the agent, the depth model has to be rotated together with the agent.

#### 2.6.2 Matched filters and depth-dependent coupling matrix for the adaptive MFA

The equations of the modified KvD algorithm form the basis of the adaptive MFA (a small constant *ɛ* is inserted in Eq (33), the equation of the inverse distance, to avoid the singularity in case ${\vec{d}}_{i}=\vec{t}$):

$$\mu_{i}=-\frac{\vec{t}\cdot \left({\vec{p}}_{i}-{\vec{d}}_{i}\times \vec{r}\right)}{1-{\left(\vec{t}\cdot {\vec{d}}_{i}\right)}^{2}+\varepsilon },$$ (33)

$$\vec{t}=\frac{-1}{\langle \mu \rangle }(\langle \vec{p}\rangle +\vec{r}\times \langle \vec{d}\rangle -\langle \mu \;\left(\vec{t}\cdot \vec{d}\right)\;\vec{d}\rangle ),$$ (34)

$$\vec{r}=\langle \vec{p}\times \vec{d}\rangle +\vec{t}\times \langle \mu \vec{d}\rangle +\langle (\vec{r}\cdot \vec{d})\;\vec{d}\rangle .$$ (35)

As shown in section 2.4 these iteration equations can be decomposed into the product of the current optical flow and a standard template, on the one side, and a coupling matrix, on the other side (see following equation). The coupling matrix is the part of the adaptive model that contains the depth values *μ*_*i*_:

$$(\begin{array}{cc} M^{tt} & M^{tr}\\ M^{rt} & M^{rr} \end{array})\cdot (\begin{array}{c} \vec{t}\\ \vec{r} \end{array})=(\begin{array}{c} \langle -\vec{p}\rangle \\ \langle \vec{p}\times \vec{d}\rangle \end{array}),$$ (36)

$$M^{tt}=\langle \mu \rangle I-\langle \mu \vec{d}⊗\vec{d}\rangle ,$$ (37)

$$M^{tr}=-\langle \vec{[d}\times ]\rangle ,$$ (38)

$$M^{rt}=\langle \mu \;[\vec{d}\times ]\rangle ,$$ (39)

$$M^{rr}=I-\langle \vec{d}⊗\vec{d}\rangle ,$$ (40)

where the matrix $\vec{[d}\times ]$ is defined by

$$\vec{[d}\times ]\vec{v}=\vec{d}\times \vec{v},$$

$$\vec{[d}\times ]=(\begin{array}{ccc} 0 & -d_{3} & d_{2}\\ d_{3} & 0 & -d_{1}\\ -d_{2} & d_{1} & 0 \end{array}),$$

i.e. the cross product of the two vectors $\vec{d}$ and $\vec{v}$ can be expressed as multiplication of matrix $\vec{[d}\times ]$ and vector $\vec{v}$.

#### 2.6.3 The case of spherically distributed flow vectors

For the special case of spherically distributed flow vectors the depth-dependent coupling matrix (see subsection (2.3.1) is given explicitly. In the simplest case of an agent being in the center of a sphere, i.e. if all inverse distances have the same value, the depth-dependent coupling matrix is proportional to the identity matrix (see end of subsection (2.3.1). In the general case, i.e. when the inverse distances can have arbitrary values, the entries of the depth-dependent coupling matrix are the expansion coefficients of the first three orders of the real valued spherical harmonic functions.

**Fig 4:**
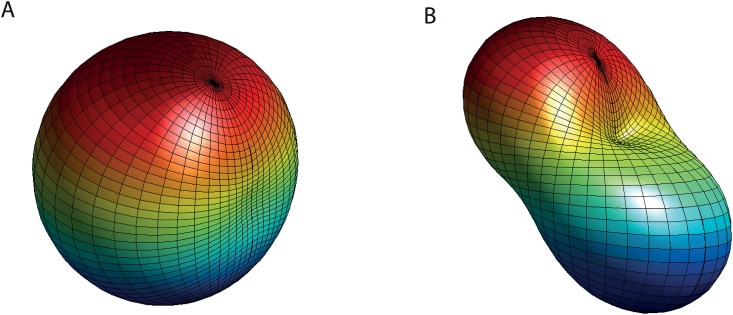
Spherical harmonic functions from the expansion of the inverse distances *μ*_*i*_. **A)** The sum of the zeroth order function and a first-order dipole-function. **B)** The sum of the zeroth order function and a second-order quadrupole-function.

Then the environmental depth structure *μ*_*i*_ can be described by a real-valued function *μ*_*ϑφ*_ parameterized by the azimuth angle *φ* and the elevation angle *ϑ* (in a spherical coordinate system). Such functions can be described by an expansion of real-valued spherical harmonic functions [36] (see appendix 5.3 and Fig 4). Lower orders of these functions contain less details than higher order functions.

Given a function *μ*(*ϑ*, *φ*) depending on azimuth angle *φ* and elevation angle *ϑ* the expansion coefficients *a*_*ln*_ are

$$a_{ln}=\int_{\varphi =0}^{2\pi }\int_{\vartheta =0}^{\pi }R_{ln}\;(\vartheta ,\varphi )\cdot \mu \;(\vartheta ,\varphi )\cdot \sin\vartheta d\vartheta d\varphi .$$ (41)

*R*_*ln*_ (*ϑ*, *φ*) represent, for example, a dipole function for specific *l* and *n*. The corresponding coefficient *a*_*ln*_ provides information about how pronounced the specific dipole part is in the expanded function *μ* (*ϑ*, *φ*).

With the help of all coefficients *a*_*ln*_ the expanded function *μ* (*ϑ*, *φ*) is given by the reverse transformation

$$\mu \;(\vartheta ,\varphi )=\sum_{l=0}^{N}\sum_{n=-l}^{+l}a_{ln}R_{ln}\;(\vartheta ,\varphi ),$$ (42)

with *N* = 0, 1, 2, ….

The terms 〈*μ*〉 and $\langle \mu \vec{d}⊗\vec{d}\rangle$ in *M* ^*tt*^ and the term $\langle \mu [\vec{d}\times ]\rangle$ in *M* ^*rt*^ are the sole terms which contain the inverse distances *μ*_*i*_. In appendix (5.3) it is shown, that $\vec{d}⊗\vec{d}$ and $\vec{d}$ can be expressed through real valued spherical harmonic functions when these term are given by their continuous counterparts ${\vec{d}}_{\vartheta \varphi }$. It can be directly seen that the continuous counterparts of $\vec{d}$ are the first-order real valued harmonic functions (except for a constant factor), whereas some transformations are needed to see that the continuous counterparts of $\vec{d}⊗\vec{d}$ are linear combinations of the zeroth and second order real valued harmonic functions.

Due to the linearity of an integral expression and the orthogonality of the spherical harmonic functions [36],

$$\int R_{ln}\;(\vartheta ,\varphi )\;R_{l^{\prime }n^{\prime }}\;(\vartheta ,\varphi )\;d\Omega =\delta_{ll^{\prime }}\delta_{nn^{\prime }},$$ (43)

the terms 〈*μ*〉, $\langle \mu \vec{d}⊗\vec{d}\rangle$ and $\langle \mu [\vec{d}\times ]\rangle$ can be interpreted as the definition equations for specific coefficients of a spherical harmonic expansion of *μ* (*ϑ*, *φ*) [see Eq (41)]. Hence, in the spherical case 〈*μ*〉, $\langle \mu \vec{d}⊗\vec{d}\rangle$ and $\langle \mu [\vec{d}\times ]\rangle$ in the coupling matrix (Eqs (38) until (40)) can be replaced by the coefficients of spherical harmonic functions:

$$M^{tt}=\sqrt{4\pi }\cdot a\cdot (I-\frac{1}{3}I)-\sqrt{\frac{4\pi }{15}}\cdot C$$

$$C=(\begin{array}{ccc} -c_{4}-\sqrt{\frac{1}{3}}\cdot c_{1} & c_{5} & c_{2}\\ c_{5} & c_{4}-\sqrt{\frac{1}{3}}\cdot c_{1} & c_{3}\\ c_{2} & c{}_{3} & \sqrt{\frac{4}{3}}\cdot c_{1} \end{array})$$

$$M^{tr}=0$$

$$M^{rt}=\sqrt{\frac{4\pi }{3}}\cdot B$$

$$B=(\begin{array}{ccc} 0 & -b_{3} & b_{2}\\ b_{3} & 0 & -b_{1}\\ -b_{2} & b_{1} & 0 \end{array})$$

$$M^{rr}=\frac{2}{3}I$$

where *a* is the coefficient of the zeroth spherical harmonic function, which cannot be determined by the algorithm as explained earlier. The three *b*’s are the three coefficients of the first-order spherical harmonic functions, the dipole functions. The five *c*’s are the five coefficients of the second-order spherical harmonic functions, the quadrupole functions.

The only non-constant parameters in the depth-dependent matrix are the nine coefficients of the expansion by spherical harmonics. The nine parameters the agent has to memorize during flight have, in the case regarded here, a physical interpretation: They are the dipole and quadrupole parts of the depth distribution of the environment. The non-existence of higher-order coefficients in the depth-dependent matrix indicates that these orders contain no information for solving the self-motion problem. If the distances are constant, the matrix *M* is the identity matrix (except for a normalization constant) as mentioned before.

The computation of the nine coefficients might not seem to be biologically plausible at first sight. However, the computation of one coefficient *a*_*ln*_ of the expansion corresponds to a weighted wide-field integration and is reminiscent of the function of LPTCs in flies [16, 17, 25, 26]. One could imagine a neuron for each of the nine parameters *a*_*ln*_, which represents a specific global property of the depth structure.

With regard to the computational effort a full inversion of a 6 × 6 matrix is not required. The submatrix *M* ^*tr*^ is zero in the spherical case, hence only an inversion of the submatrix *M* ^*tt*^ is required. If the quadrupoles in this submatrix are sufficiently small, the inverse matrix can be linearly approximated by a Neumann series [37], (*I* − *A*)^−1^ = *I* + *A* + *A* ^2^ + *A* ^3^ + ….

#### 2.6.4 Test of the adaptive MFA

In this section we compare quantitatively the adaptive and the original MFA. We present a simulation in a very simple environment where the agent moves inside a sphere (see Fig 3 and methods 4.4). Nothing is known in advance about the flight directions and whereabouts of the agent inside the sphere. Hence, the covariance matrices of the original MFA are set proportional to the unit matrix. The chosen trajectory in this setting is a sinusoidal curve. On this trajectory the current depth distribution differs essentially from that in the center of the sphere (Fig 3A).

The amount of translation and rotation in each step varies along the trajectory. The steps are chosen so that the maximum rotation angle is about 8 degrees per step ensuring that the approximation of the KvD algorithm, which is valid for small rotation angles, still holds. The maximum rotation angles between the processed images is in the same range as the maximum rotation angles during saccades of flying insects [38, 39].

**Fig 5:**
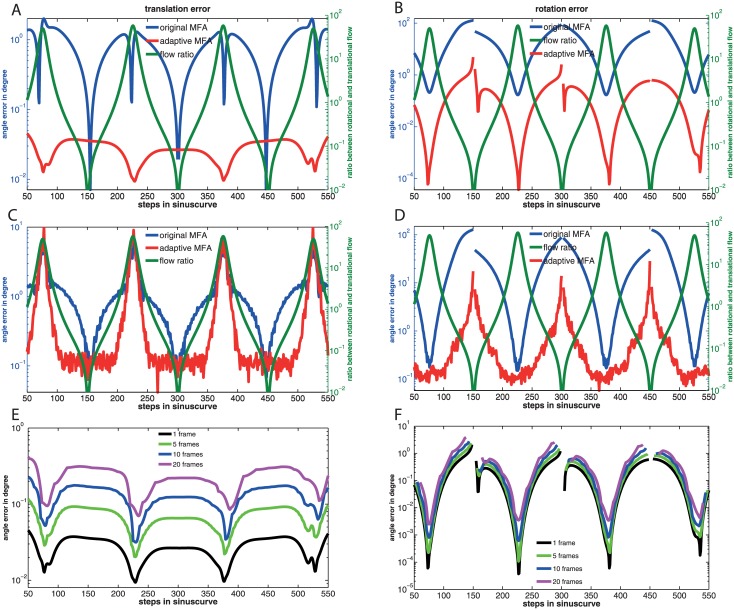
As in Fig 2 an agent flies inside a sphere (see methods 4.4). The trajectory is a sinus curve with two full periods. The amplitude of the sinus curve is 0.5 of the radius of the sphere. The sinus curve is lifted in the sphere by 0.3 units in z-direction to avoid symmetries in the depth model. The agent performs 600 steps which result in rotation angles of up to 8 degrees per frame. The initialization phase is not shown. The left figures shows the error of the translation estimates and the right figures shows the error of the rotation estimates. The y-axes show the angle error $\text{arccos}\left(\frac{{\vec{\theta }}^{\text{est}}\cdot \vec{\theta }}{|{\vec{\theta }}^{\text{est}}|\cdot |\vec{\theta }|}\right)$, between the estimated self-motion axis of ${\vec{\theta }}^{\text{est}}$ and the axis of the true self-motion values $\vec{\theta }$. The estimated angle errors have a pole, when the rotation gets zero at the inflection points of the sinusoidal curve (see methods 4.4). The small region around the poles are cut out in the figures. **A, B)** The two figures show the error of the adaptive MFA (red curve) and the original MFA (blue curve) with a constant inverse distant assumption for the original MFA. The adaptive MFA is updated every time step. The right y-axes of the figures show the averaged ratio between the rotational and translational optic flow. **C, D)** shows the adaptive MFA and the original MFA as in figures A and B, but with an error of 10% added to the optical flow. **E, F)** show different update frequencies of the depth model. All models rotate with the agent. The update frequencies are: black = 1 frame, green = 5 frames, blue = 10 frames and violet = 20 frames.

On the whole, the adaptive MFA performs better than the original non-adaptive MFA (Fig 5). The second y-axes on the right side of the figure show the averaged ratio between the rotational and translational optic flow at every time step. Due to the sinusoidal trajectory of the agent this ratio varies between a factor of hundred in favor of the rotational or translational optic flow. When an optic flow component is overlayed hundredfold by the other flow component the coupling terms $\vec{r}\times \langle \vec{d}\rangle$ and $\vec{t}\times \langle \mu \vec{d}\rangle$ between the flow components are the dominant parts in the related estimation Eqs (34) and (35). For a spherical field of view the term $\vec{r}\times \langle \vec{d}\rangle$ is zero. But the term $\vec{t}\times \langle \mu \vec{d}\rangle$ depends on the dipole components of the inverse distance *μ* (see appendix 5.3.1). Hence, only the rotation estimate is affected by errors in the estimated dipoles. Because the original MFA does not determine the values for the dipole components of the inverse distance for the current optical flow, the rotation estimates become totally useless, whenever the translational flow component is dominant. The angle error between the estimated rotation axis and the true rotation axis increases to a value of about hundred degrees. Whereas the angle error of the adaptive MFA does not exceed few degrees.

If an Gaussian error is added to the optical flow with a standard deviation of ten percent of the averaged overall flow value (Fig 5), one could expect that a translational or rotational flow component, which is overlayed hundredfold by the other flow component disappears totally in this flow error, because the error is tenfold higher as the flow component in this situation. But the estimators use hundreds or a few thousands of flow vectors to estimate self-motion. Due to the large number of flow vectors (insects usually have between a few hundred (e.g. fruit fly) and a few thousand ommatidia (bee, dragonfly) per eye), the self-motion can be still estimated in this case within a useful error range. The results are shown for about 5000 flow vectors (Fig 5). The error increases for both the translational and the rotational self-motion estimate to a value of about 10 degrees. This error is additive to the error described in the upper panels of Fig 5 and affects the adaptive MFA in the same way as the original MFA.

In the bottom panels of Fig 5 different update rates are tested. Even for an update at only every twentieth optical flow processing step the errors remain in a useful range.

Albeit the simplicity of the simulation it shows some basic features of the compared algorithms. The simulation does not generate any outliers due to moving objects or depth discontinuities. A small number of outliers will not affect the MFA as a consequence of the linear summation over thousands of optic flow vectors. More complex simulations in virtual environments with rendered images and EMDs is left to future work (Strübbe et al., in prep.).

## 3 Discussion

The aim of this study is to develop an adaptive matched filter approach to self-motion estimation which could be in principle the underlying concept of self-motion estimation in flying insects. As a novel characteristic, this approach assumes an adaptation to the depth structure in the insect visual motion pathway, an assumption that is supported by recent experimental evidence [31, 40, 41]. Our approach starts from a theoretical point of view by analysing and unifying the non-adaptive matched filter approach (MFA) to self-motion estimation together with the Koenderink van Doorn (KVD) algorithm which incorporates an estimation of the depth values.

To take advantage of both algorithms, some mathematical problems had to be solved. First, it was shown that the two algorithms are equivalent in case the distances to objects in the environment are assumed to be known. Secondly, a bias in the KvD algorithm was removed by a small correction of the iteration equations. And last but not least, an analysis of the specific case of a spherical field of view, reminiscent of that of flying insects, shows that the depth structure can be represented by only eight parameters without losing relevant information for self-motion estimation and that these eight parameters are the dipole and quadrupole moments of the spherical harmonics.

Technical and biological systems have different origins and often operate under different conditions. Biological systems arise through evolutionary adaptation. They usually have to operate in a great variety of environments. Hence, the neural computations underlying the animal’s behavior need to be particularly robust. In addition, the animal has restrictions with respect to its computational hardware. Neuronal circuits can perform linear transformations in parallel by a dendritic weighted summation of the inputs of a neuron and non-linear operations through the non-linear response behavior of a neuron to its overall input. Nonetheless, a non-linear operation, such as computing the inverse of a matrix, with changing entries, is not easy to implement by neuronal hardware. The bio-inspired computational model analyzed here is the MFA of self-motion estimation. It is a linear model for a fixed depth assumption, derived under the side condition of maximal robustness against errors in the measured optical flow field (see Eq (5) from Franz et al. [11]).

The KvD algorithm of self-motion estimation which is compared with the MFA model was derived analytically in a technical framework on the basis of a minimization principle. The resulting iteration equations represent a gradient descent where the current self-motion parameters are used to determine a better depth model and the new depth model is used to determine a better estimate of self-motion in the next iteration step. If one considers only one iteration step, where the depth model is seen as fixed, the self-motion estimation is linear. It is not only linear, but also equivalent to the biologically inspired MFA which uses a fixed depth distribution.

The equivalence of both models becomes evident within the framework of linear estimator theory. There exists an unique optimal estimator for a linear estimation problem with error-prone inputs. The Gauss-Markov theorem describes this estimator. Both compared methods represent this optimal solution.

However, some differences exists. In the MFA Franz et al. [11] weighted the filters by matrices that represent additional assumptions about the situation under which the self-motion is estimated. If these assumptions are correct, the weighting of the filters improves self-motion estimation; however, if these assumptions are incorrect in the current situation, the estimator gets worse. Hence, the additional matrices make the estimator more specific. These matrices can also be implemented in the KvD algorithm by a modification of the minimization principle. When, for example, it is known, that the optical flow can be measured more accurately below the horizon, because the objects are generally closer there, this knowledge can be taken into account by introducing weights in the initial equation. We argue that it is not always useful to take knowledge about the preferred self-motion directions into account. Even when the moving agent solely translates in the forward direction a disturbance can lead to a passive translation also in other directions.

From a mathematical point of view the bias of the KvD algorithm is remarkable. When the depth distribution of the environment has to be determined together with the self-motion parameters, the estimation problem is no longer linear. The standard procedure for estimating parameters from inputs, which are disturbed by Gaussian errors, is the minimization of the mean squared error. It might be counter-intuitive that the true self-motion values are not even a local minimum. The standard approach fails, because the standard condition assumes that an increasing number of measured values, here the flow vectors, are accompanied by a fixed number of estimation parameters. However, for the non-linear estimation problem the number of distance values increases together with the number of flow vectors. Only the number of self-motion parameters remains constant. With every additional flow vector additional information about the self-motion parameters is obtained, because one gets two additional independent values from the flow vector, but only one additional parameter has to be estimated (the distance corresponding to the flow vector). However, the standard approach does not use this additional information in an optimal way.

Here we derived a modified version of the KvD algorithm that is not derived from a minimization principle. Hence it is not clear whether it leads to the best estimate for a given finite number of flow vectors. Rather, the numerical simulation indicates that the modified version has the desired property that the algorithm converges to the real self-motion values for an infinite number of flow vectors. It is left to further mathematical work to analyze optimal criteria for the non-linear estimation problem in case of a finite number of flow vectors.

Based on this modified version of the KvD algorithm an adaptive MFA was derived. It was shown that it is a critical issue to correctly determine the dipole components. If a small rotation is superimposed by a large translation the non-adaptive MFA cannot provide useful rotation estimates, whereas the adaptive MFA is accurate up to a few degrees. A situation where a relatively large translation encounters a relatively small rotation is given in the inter-saccadic phases of insect flight [38, 39, 42]. In these phases the insect tries to avoid any rotation. If the insect stabilizes its flight with the help of the visual system, the non-adaptive MFA cannot be the underlying concept to detect small rotations in these phases in environments it is not tuned to. To estimate rotations, which are superimposed by a large translation, one has to determine the current dipole components, as is done by the adaptive MFA.

The adaptive MFA was inspired by the finding that for a spherical field of view the depth structure of the environment can be represented by only eight parameters without losing relevant information for self-motion estimation and by the fact that the visual system of insects has an almost spherical field of view. The spherical field of view is also a desirable property of technical systems which are designed to estimate their self-motion on the basis of optical flow fields. Such systems can be realized by panoramic cameras [43, 44].

Adaptation to the depth structure of the environment means that the adaptation takes place on another time scale than the image processing itself. Hence, some information about the depth structure has to be memorized by the system. The result that exact self-motion can only be estimated for a spherical field of view, if eight parameters about the depth structure of the environment are known, is therefore in accordance with the limited computational resources of insects.

Motion adaptation was analyzed in the insect visual pathway and found to depend on the overall velocity in the visual field [27, 31, 32, 40, 41, 45]. Since, at least during translational motion, the overall retinal velocity depends on the depth distribution of the environment. The experimentally characterized processes of motion adaptation may well play a role in an adaptive mechanism of self-motion estimation as proposed in the present study. Here we give a short analysis from a theoretical point of view which components are needed for the adaptive MFA. Minimalistically, one needs eight model neurons for the eight depth parameters. The weighted summation over the inputs of one of these model neurons corresponds to one of the eight integrals over the depth distribution, where the spherical harmonic functions play the role of the weighting parameters. Examples of neurons performing such an integration are the LPTC neurons of flies, the neuronal candidates for the six model neurons, which represent the matched filters for the six self-motion components. Given the properties of LPTCs [16, 17, 24–26], it is likely that one hypothetical model neuron for depth representation does not cover the whole sphere. Due to the linearity of self-motion estimation LPTCs can be combined to represent one self-motion component. On this basis, it might be possible that one LPTC codes information for both translation and rotation when the corresponding flow fields resemble each other within the receptive field of the neuron. Hence, the hypothetical depth neurons could be realized by a network of neurons with each neuron receiving input from only part of the visual field.

The hypothetical neurons representing the depth structure need some pre- and post-processing. In the adaptive MFA, only the pre-processing contains non-linear operations, namely the transformation of the optical flow into local depth values that are integrated afterwards by the hypothetical model neurons. One can assume that the determination of the depth values is simplified during nearly pure translation as is characteristic of the insect saccadic flight strategy [19]. In these phases the depth structure can be determined more easily, because the optic flow is not superimposed by a rotational component [46, 47].

The post-processing concerns the determination of the depth-dependent matrix which corrects the outputs of the six model neurons, corresponding to the motion sensitive LPTC cells. From a mathematical point of view, two subsequent linear transformations can be combined in a single linear transformation. In the adaptive MFA we have two subsequent linear transformations: the fixed linear transformation by the six model neurons that receive direct optical flow input and the adaptive linear transformation by the depth-dependent matrix, the entries of which are the responses of the eight depth neurons. There are two options where the adaptation could take place: The two linear transformations could be merged into one linear transformation, which means that the adaptation takes place at an early stage of optic flow processing. Alternatively, one could assume that the two linear transformations are spatially separated, and the depth-dependent matrix is realized by an adapting linear circuitry, which wires the early stage neurons.

The linear transformation given by the depth dependent matrix can be obtained without a matrix inversion by applying an appropriate linearization of the inverse depth dependent matrix (see section 2.6.3). With this simplification and the above simplification of depth capturing the adaptive MFA can be realized by relatively simple neuronal circuits.

## 4 Materials and Methods

### 4.1 Numerical test and simulation

We used a numerical test (Fig 1) and a simulation (Fig 5) to show the performance of the considered self-motion estimators based on optical flow fields. The optical flow fields used here are computed in all cases from the flow Eq (1) where the distances, the viewing directions and the self-motion parameters are given. In some cases a Gaussian error was added to the optical flow values. The task of the self-motion estimators is to determine the self-motion parameters from these flow fields.

Whereas the distances in the numerical test are obtained by a random process, the distances in the simulation are defined by the environment. In the numerical test the individual estimates are independent of each other. In the simulation a trajectory through the environment is constructed to enable an agent to use the depth model of the environment for a series of estimates at subsequent trajectory points.

For programming and testing the algorithm the programming language Matlab was used.

### 4.2 Construction of a spherical field of view

For the simulation and the numerical test a spherical visual field of view is needed. It is not a trivial task to arrange a number of viewing directions on a sphere in a way that the density is equally distributed. Here we used an iterative solution. The iteration starts with a Platonic solid, an octahedron. An octahedron has eight faces: Eight equilateral triangles with the same side lengths, which surround a symmetric solid.

In each iteration step every triangle is replaced by four new triangles with one triangle placed in the middle of the old triangle and the other three are placed in corners. The mid-points of the sides of the old triangle coincide with the corners of the new triangles. These mid-points are projected radially on the surface of the sphere which surround the object so that all corners of the new triangles lie on this sphere.

In each iteration step the sphere is covered with nearly identical triangles. After the last iteration step the mid-points of each triangle give a viewing directions. With an arbitrary number of iterations *n* the number of viewing directions are 8 ⋅ 4^*n*^.

### 4.3 Numerical test of the bias of the KvD algorithm

In the numerical test of the bias of the KvD algorithm two configurations are tested, one with a spherical field of view and one with a non-spherical field. The non-spherical field of view is realized by the same procedure as in the spherical case (see methods 4.2), but two starting triangles are left out. The omitted triangles are opposite triangles both placed in the upper part of the sphere. This configuration has some obvious symmetry, but avoids the case that each omitted viewing direction has an omitted counterpart on the other side of the sphere.

The number of viewing directions is increased by increasing the number of iterations starting from an octahedron. Each step increases the number of viewing directions by four. For each number of viewing directions 40 self-motion estimation trials are tested.

### 4.4 Simulation of the adaptive MFA

The environment used for the simulation is a unit sphere in which the agent flies on a sinusoidal trajectory. The effect of adaptation should be larger if, for example, the agent flies outdoors and then enters a tunnel which is a common experimental set-up for navigation studies in flying insects [48–51]. However, the test of the adaptive MFA in this kind of environment needs a 3-d engine for rendering images, on which the flow vectors are estimated with motion detectors like the Reichardt detector or the Lucas-Kanade detector. This issue will be addressed in another study (Strübbe et al., in prep.).

For the trajectory a sinusoidal curve was chosen (see Fig 3 right). Although it is typical that flying insects use a saccadic flight strategy to separate translation and rotation, the self-motion estimators in this study were analyzed under the condition of a combined translation and rotation. In each run the agent flies along the trajectory and the angle error between the true self-motion axes and the estimated axes of the tested estimators are shown in Fig 5.

## 5 Appendix

### 5.1 Derivation of the equivalence of the MFA and KvD algorithm

From Eq (17) one obtains together with the equations for the coupling matrix Eqs (21), (22) and (23) and the definitions of the templates Eqs (13) and (14):

$$\vec{a}=\hat{M}\;\cdot \;\vec{\theta }=\left(\begin{array}{cc} M^{tt} & M^{tr}\\ M^{rt} & M^{rr} \end{array}\right)\;\cdot \;\left(\begin{array}{c} \vec{t}\\ \vec{r} \end{array}\right)=\left(\begin{array}{c} \langle {\vec{T}}_{1}^{t}\cdot \vec{p}\rangle \\ \langle {\vec{T}}_{2}^{t}\cdot \vec{p}\rangle \\ \langle {\vec{T}}_{3}^{t}\cdot \vec{p}\rangle \\ \langle {\vec{T}}_{1}^{r}\cdot \vec{p}\rangle \\ \langle {\vec{T}}_{2}^{r}\cdot \vec{p}\rangle \\ \langle {\vec{T}}_{3}^{r}\cdot \vec{p}\rangle \end{array}\right)$$ (44)

$${\left(M^{tt}\right)}_{ab}=\langle \mu^{2}\rangle {\vec{e}}_{a}\cdot {\vec{e}}_{b}-\langle \mu^{2}\;\left({\vec{e}}_{a}\cdot \vec{d}\right)\cdot \left({\vec{e}}_{b}\cdot \vec{d}\right)\rangle$$ (45)

$${\left(M^{tr}\right)}_{ab}=\langle \mu \;({\vec{e}}_{a}\times {\vec{e}}_{b})\cdot \vec{d}\rangle$$ (46)

$${\left(M^{rt}\right)}_{ab}=-\langle \mu \;({\vec{e}}_{a}\times {\vec{e}}_{b})\cdot \vec{d}\rangle$$ (47)

$${\left(M^{rr}\right)}_{ab}={\vec{e}}_{a}\cdot {\vec{e}}_{b}-\langle ({\vec{e}}_{a}\cdot \vec{d})\cdot ({\vec{e}}_{b}\cdot \vec{d})\rangle$$ (48)

$${\vec{T}}_{ia}^{t}=-\mu_{i}\left({\vec{d}}_{i}\times {\vec{e}}_{a}\times {\vec{d}}_{i}\right)$$ (49)

$${\vec{T}}_{ia}^{r}=-{\vec{e}}_{a}\times {\vec{d}}_{i}$$ (50)

Multiplying $\vec{t}$ and $\vec{r}$ with the matrices and $\vec{p}$ with the templates leads to the two equations:

$$\begin{array}{ll} {\left(M^{tt}\cdot \vec{t}+M^{tr}\cdot \vec{r}\right)}_{a}= & \langle \mu^{2}\rangle t_{a}-\langle \mu^{2}\underset{b}{\sum }\left({\vec{e}}_{a}\cdot \vec{d}\right)\cdot \left({\vec{e}}_{b}\cdot \vec{d}\right)\cdot t_{b}\rangle \\ & +\langle \mu \underset{b}{\sum }\left(\left({\vec{e}}_{a}\times {\vec{e}}_{b}\right)\cdot \vec{d}\right)\cdot r_{b}\rangle \end{array}$$ (51)

$$=\langle {\vec{T}}_{a}^{t}\cdot \vec{p}\rangle =\langle \mu \vec{p}\cdot (-\vec{d}\times {\vec{e}}_{a}\times \vec{d})\rangle$$ (52)

$$\begin{array}{ll} {\left(M^{rt}\cdot \vec{t}+M^{rr}\cdot \vec{r}\right)}_{a}= & -\langle \mu \underset{b}{\sum }\left(\left({\vec{e}}_{a}\times {\vec{e}}_{b}\right)\cdot \vec{d}\right)\cdot r_{b}\rangle \\ & +t_{a}-\langle \underset{b}{\sum }\left({\vec{e}}_{a}\cdot \vec{d}\right)\cdot \left({\vec{e}}_{b}\cdot \vec{d}\right)\cdot t_{b}\rangle \end{array}$$ (53)

$$=\langle {\vec{T}}_{a}^{r}\cdot \vec{p}\rangle =\langle \vec{p}\cdot (\vec{d}\times {\vec{e}}_{a})\rangle$$ (54)

The term $\sum_{b}{\left[\left({\vec{e}}_{a}\cdot \vec{d}\right)\cdot \left({\vec{e}}_{b}\cdot \vec{d}\right)\right]}_{ab}\cdot v_{b}$ with an arbitrary vector $\vec{v}$ can be expressed through the dyadic product $\sum_{b}{\left[\vec{d}⊗\vec{d}\right]}_{ab}\cdot v_{b}=\left(\vec{v}\cdot \vec{d}\right)d_{a}$, and the term $\sum_{b}\left(\left({\vec{e}}_{a}\times {\vec{e}}_{b}\right)\cdot \vec{d}\right)\cdot v_{b}$ can be transformed with the triple product rule into

$$\begin{array}{ccc} \sum_{b}\;(({\vec{e}}_{a}\times {\vec{e}}_{b})\cdot \vec{d})\cdot v_{b} & = & (\vec{d}\times {\vec{e}}_{a})\cdot \sum_{b}{\vec{e}}_{b}\cdot v_{b}=(\vec{d}\times {\vec{e}}_{a})\cdot \vec{v}\\ & = & (\vec{v}\times \vec{d})\cdot {\vec{e}}_{a}={\left[\vec{v}\times \vec{d}\right]}_{a} \end{array}$$ (55)

The right side of the Eq (52), $\langle \mu \vec{p}\cdot \left(-\vec{d}\times {\vec{e}}_{a}\times \vec{d}\right)\rangle$, is equal to $\langle \mu \vec{p}\cdot \left(-{\vec{e}}_{a}+\left({\vec{e}}_{a}\cdot \vec{d}\right)\vec{d}\right)\rangle =\langle -\mu \vec{p}\cdot {\vec{e}}_{a}\rangle =-{\left[\langle \mu \vec{p}\rangle \right]}_{a}$, because $\vec{p}$ and $\vec{d}$ are orthogonal. With the triple product identity $\langle \vec{p}\cdot \left(\vec{d}\times {\vec{e}}_{a}\right)\rangle$ in Eq (54) is transformed into $\langle {\vec{e}}_{a}\cdot \left(\vec{p}\times \vec{d}\right)\rangle ={\left[\langle \vec{p}\times \vec{d}\rangle \right]}_{a}$.

Writing the *a* indexed values as vectors, we obtain

$$\langle \mu^{2}\rangle \vec{t}-\langle \mu^{2}(\vec{t}\cdot \vec{d})\;\vec{d}\rangle +\vec{r}\times \langle \mu \vec{d}\rangle =-\langle \mu \vec{p}\rangle$$

$$-\vec{t}\times \langle \mu \vec{d}\rangle +\vec{r}-\langle (\vec{r}\cdot \vec{d})\;\vec{d}\rangle =\langle \vec{p}\times \vec{d}\rangle ,$$

which results in the iteration Eqs (11) and (12) of the KvD algorithm:

$$\vec{t}=-\frac{1}{\langle \mu^{2}\rangle }\{\langle \mu \vec{p}\rangle +\vec{r}\times \langle \mu \vec{d}\rangle -\langle \mu^{2}\left(\vec{t}\cdot \vec{d}\right)\vec{d}\rangle \},$$

$$\vec{r}=\langle \vec{p}\times \vec{d}\rangle +\vec{t}\times \langle \mu \vec{d}\rangle +\langle (\vec{r}\cdot \vec{d})\;\vec{d}\rangle ,$$

where $\frac{1}{\langle \mu^{2}\rangle }$ represents the Lagrange multiplier *ξ*.

### 5.2 Bias-term of the KvD algorithm

The iteration Eqs (10), (11) and (12) do not converge to the true parameters ${\vec{t}}^{0}$, ${\vec{r}}^{0}$ and $\mu_{i}^{0}$ in general, even in the limit of an infinite number of flow vectors. We consider unbiased and independently distributed flow vector errors, $E\;\left(\Delta {\vec{p}}_{i}\right)=0$, $\text{cov}\;\left(\Delta {\vec{p}}_{i},\Delta {\vec{p}}_{j}\right)=0$ if *i* ≠ *j*. Their variance $\text{var}\left(\Delta {\vec{p}}_{i}\right)$ may depend on the direction *i*.

The mean of an infinite number of independent flow vector errors converges to the mean of the expectation value,

$${\langle \Delta {\vec{p}}_{i}\rangle }_{\infty }=0,$$ (56)

for an arbitrary integration area around viewing direction *i*. Because the error vectors are independent from each other, the mean of the product of the error vectors and an arbitrary direction dependent vector function ${\vec{f}}_{i}$ is zero,

$${\langle {\vec{f}}_{i}\cdot \Delta {\vec{p}}_{i}\rangle }_{\infty }=0.$$ (57)

To show that the KvD algorithm does not converge to the real values for an infinite number of flow vectors, we start by assuming the opposite. If the KvD algorithm had a minimum at the true values ${\vec{t}}^{0}$, ${\vec{r}}^{0}$ and $\mu_{i}^{0}$ we could insert these values in the iteration Eqs (10), (11) and (12) and would get the same values back. Substituting the true values for translation and rotation in the equation for the nearness Eq (10), we get the nearness error △*μ*_*i*_ which directly depends on the flow vector errors:

$$\Delta \mu_{i}=-\frac{{\vec{t}}^{0}\cdot \Delta {\vec{p}}_{i}}{1-{\left({\vec{t}}^{0}\cdot {\vec{d}}_{i}\right)}^{2}}.$$

The estimated translation after an infinite number of iterations $\vec{t}(\infty )={\text{lim}}_{n\to \infty }\vec{t}(n)$ shows the following equation. Please note that we consider two limits: the limit of infinite iteration steps, lim_*n* → ∞_, and the limit of an infinite number of flow vectors, indicated by an infinity symbol as index of the brackets which stands for the mean over all viewing directions.

$$\begin{array}{lll} \vec{t}\left(\infty \right) & = & {\vec{t}}^{0}+\Delta \vec{t}=-\xi \{{\langle \mu \vec{p}\rangle }_{\infty }+{\vec{r}}^{0}\times {\langle \mu \vec{d}\rangle }_{\infty }-{\langle \mu^{2}({\vec{t}}^{0}\cdot \vec{d})\vec{d}\rangle }_{\infty }\}\\ & = & -\xi \{{\langle \left(\mu^{0}+\Delta \mu \right)\left({\vec{p}}^{0}+\Delta \vec{p}\right)\rangle }_{\infty }+{\vec{r}}^{0}\times {\langle \left(\mu^{0}+\Delta \mu \right)\vec{d}\rangle }_{\infty }-{\langle {\left(\mu^{0}+\Delta \mu \right)}^{2}({\vec{t}}^{0}\cdot \vec{d})\vec{d}\rangle }_{\infty }\}. \end{array}$$

If $\vec{t}(\infty )$ is a stable minimum $△\vec{t}$ must vanish:

$$\begin{array}{lll} △\vec{t} & = & -\xi \;\left\{{\langle \mu^{0}\Delta \vec{p}+△\mu {\vec{p}}^{0}+△\mu \Delta \vec{p}\rangle }_{\infty }+{\vec{r}}^{0}\times {\langle △\mu \vec{d}\rangle }_{\infty }-{\langle (2\mu^{0}△\mu +△\mu^{2})\;\left({\vec{t}}^{0}\cdot \vec{d}\right)\;\vec{d}\rangle }_{\infty }\right\}\\ & = & -\xi \;\left\{{\langle △\mu \Delta \vec{p}\rangle }_{\infty }-{\langle △\mu^{2}\left({\vec{t}}^{0}\cdot \vec{d}\right)\;\vec{d}\rangle }_{\infty }\right\}\\ & = & \xi \;\left\{{\langle \frac{{\vec{t}}^{0}\cdot \Delta \vec{p}}{1-{\left({\vec{t}}^{0}\cdot \vec{d}\right)}^{2}}\Delta \vec{p}\rangle }_{\infty }+{\langle \frac{\left({\vec{t}}^{0}\cdot \Delta \vec{p})(\Delta \vec{p}\cdot {\vec{t}}^{0}\right)}{{\left(1-{\left({\vec{t}}^{0}\cdot \vec{d}\right)}^{2}\right)}^{2}}\left({\vec{t}}^{0}\cdot \vec{d}\right)\;\vec{d}\rangle }_{\infty }\right\}\\ & = & \xi \;\left\{{\langle \frac{\Delta \vec{p}⊗\Delta \vec{p}}{1-{\left({\vec{t}}^{0}\cdot \vec{d}\right)}^{2}}\rangle }_{\infty }{\vec{t}}^{0}+{\langle \frac{{\vec{t}}^{0}\cdot (\Delta \vec{p}⊗\Delta \vec{p})\cdot {\vec{t}}^{0}}{{\left(1-{\left({\vec{t}}^{0}\cdot \vec{d}\right)}^{2}\right)}^{2}}(\vec{d}⊗\vec{d})\rangle }_{\infty }{\vec{t}}^{0}\right\} \end{array}$$

For the analysis of the conditions under which the term $△\vec{t}$ vanishes, see the text (subsection 2.5.1).

### 5.3 Expression of $\vec{d}$ and $\vec{d}⊗\vec{d}$ by spherical harmonics

The vector $\vec{d}$ and the dyadic product $\vec{d}⊗\vec{d}$ are expressed by linear combinations of real-valued spherical harmonics, which are itself linear combinations of complex spherical harmonics*Y*_*lm*_,

$$R_{lm}=\{\begin{array}{cc} \frac{1}{\sqrt{2}}(Y_{lm}+{\left(-1\right)}^{m}\;Y_{l(-m)}) & m>0\\ Y_{l0} & m=0\\ \frac{1}{\sqrt{2i}}(Y_{l(-m)}-{\left(-1\right)}^{m}\;Y_{lm}) & m<0 \end{array}$$ (58)

The real-valued spherical harmonics from zero order to second-order are:

**Zeroth-Order**

$$g_{0}=\sqrt{\frac{1}{4\pi }}$$ (59)

**First-order**

$$f_{1}=\sqrt{\frac{3}{4\pi }}\sin\vartheta \cos\varphi \propto \sin\vartheta \cos\varphi$$ (60)

$$f_{2}=\sqrt{\frac{3}{4\pi }}\sin\vartheta \sin\varphi \propto \sin\vartheta \sin\varphi$$ (61)

$$f_{3}=\sqrt{\frac{3}{4\pi }}\cos\vartheta \propto \cos\vartheta$$ (62)

**Second-order**

$$h_{1}=\sqrt{\frac{5}{16\pi }}(3\cos^{2}\vartheta -1)\propto 3\cos^{2}\vartheta -1$$ (63)

$$h_{2}=\sqrt{\frac{15}{4\pi }}\sin\vartheta \cos\vartheta \cos\varphi \propto \sin\vartheta \cos\vartheta \cos\varphi$$ (64)

$$h_{3}=\sqrt{\frac{15}{4\pi }}\sin\vartheta \cos\vartheta \sin\varphi \propto \sin\vartheta \cos\vartheta \sin\varphi$$ (65)

$$h_{4}=\sqrt{\frac{15}{16\pi }}\sin^{2}\vartheta \cos(2\varphi )\propto \sin^{2}\vartheta \cos(2\varphi )$$ (66)

$$h_{5}=\sqrt{\frac{15}{16\pi }}\sin^{2}\vartheta \sin(2\varphi )\propto \sin^{2}\vartheta \sin(2\varphi )$$ (67)

#### 5.3.1 Expression of $\vec{d}$ through spherical harmonics

The components of $\vec{d}$ correspond to the first-order real-valued spherical harmonics *f*_*i*_ (*i* = 1, 2, 3). This can be seen when the components of $\vec{d}$ are written in a spherical coordinate system:

$$\vec{d}=(\begin{array}{c} d_{1}\\ d_{2}\\ d_{3} \end{array})=(\begin{array}{c} \sin\vartheta \cos\varphi \\ \sin\vartheta \sin\varphi \\ \cos\vartheta \end{array})=(\begin{array}{c} \sqrt{\frac{4\pi }{3}}\cdot f_{1}\\ \sqrt{\frac{4\pi }{3}}\cdot f_{2}\\ \sqrt{\frac{4\pi }{3}}\cdot f_{3} \end{array}).$$ (68)

The components *d*_1_, *d*_2_ and *d*_3_ are equal in this arrangement to the first-order functions *f*_1_, *f*_2_ and *f*_3_ except of a normalization factor.

#### 5.3.2 Expression of $\vec{d}⊗\vec{d}$ through spherical harmonics

First the dyadic product $\vec{d}⊗\vec{d}$ is formulated in a spherical coordinate system

$$\begin{array}{l} \begin{array}{ccc} \vec{d}⊗\vec{d} & = & \left(\begin{array}{c} d_{1}\\ d_{2}\\ d_{3} \end{array}\right)⊗\left(\begin{array}{c} d_{1}\\ d_{2}\\ d_{3} \end{array}\right)=\left(\begin{array}{ccc} d_{1}^{2} & d_{1}d_{2} & d_{1}d_{3}\\ d_{2}d_{1} & d_{2}^{2} & d_{2}d_{3}\\ d_{3}d_{1} & d_{3}d_{2} & d_{3}^{2} \end{array}\right) \end{array}\\ =\left(\begin{array}{ccc} {\text{sin}}^{2}\vartheta \;{\text{cos}}^{2}\varphi & {\text{sin}}^{2}\vartheta \;\text{sin}\varphi \text{cos}\varphi & \text{sin}\vartheta \;\text{cos}\vartheta \;\text{cos}\varphi \\ {\text{sin}}^{2}\vartheta \;\text{sin}\varphi \text{cos}\varphi & {\text{sin}}^{2}\vartheta \;{\text{sin}}^{2}\varphi & \text{sin}\vartheta \;\text{cos}\vartheta \;\text{sin}\varphi \\ \text{sin}\vartheta \;\text{cos}\vartheta \;\text{cos}\varphi & \text{sin}\vartheta \;\text{cos}\vartheta \;\text{sin}\varphi & {\text{cos}}^{2}\vartheta \; \end{array}\right). \end{array}$$ (69)

The following analysis shows that the off-diagonal elements of this matrix can each be expressed by a single second-order real-valued harmonic function, whereas the diagonal elements are linear combinations of the zeroth-order and several second-order real-valued spherical harmonic functions.

**Off-diagonal elements.** The element *d*_1_ *d*_3_ is proportional to the function *h*_2_ and the element *d*_2_ *d*_3_ is proportional to the function *h*_3_.

$$d_{1}d_{3}=\sqrt{\frac{4\pi }{15}}\cdot h_{2}$$

$$d_{2}d_{3}=\sqrt{\frac{4\pi }{15}}\cdot h_{3}$$

To see that the element *d*_1_ *d*_2_ is proportional to the function *h*_5_, *h*_5_ must be rearranged,

$$h_{5}=\sqrt{\frac{15}{16\pi }}\cdot \sin^{2}\vartheta \sin(2\varphi )=\sqrt{\frac{15}{4\pi }}\cdot \sin^{2}\vartheta \sin\varphi \cos\varphi ,$$ (70)

$$d_{1}d_{2}=\sqrt{\frac{4\pi }{15}}\cdot h_{5},$$ (71)

via the addition theorem: sin (2*x*) = 2 ⋅ sin(*x*)cos(*x*).

**Diagonal elements.** The diagonal element $d_{3}^{2}={\text{cos}}^{2}\vartheta$ can be expressed by a proper linear combination of $h_{1}=\sqrt{\frac{5}{16\pi }}\cdot \left(3{\text{cos}}^{2}\vartheta -1\right)=\sqrt{\frac{45}{16\pi }}\cdot {\text{cos}}^{2}\vartheta -\sqrt{\frac{5}{16\pi }}$ and $g_{0}=\sqrt{\frac{1}{4\pi }}$, in such a way that the constant in *h*_1_ compensate the constant in *g*_0_.

$$h_{1}+\sqrt{\frac{5}{4}}\cdot g_{0}=\sqrt{\frac{45}{16\pi }}\cdot {\text{cos}}^{2}\vartheta$$

$$d_{3}^{2}=\sqrt{\frac{16\pi }{45}}\cdot (h_{1}+\sqrt{\frac{5}{4}}\cdot g_{0})=\sqrt{\frac{16\pi }{45}}\cdot h_{1}+\sqrt{\frac{4\pi }{9}}\cdot g_{0}$$

For the expression of $d_{1}^{2}$ and $d_{2}^{2}$ the function *h*_4_ needs a closer examination. With cos (2*x*) = cos^2^ *x* − sin^2^ *x* the function *h*_4_,

$$h_{4}=\sqrt{\frac{15}{16\pi }}\sin^{2}\vartheta \cos(2\varphi )=\sqrt{\frac{15}{16\pi }}\sin^{2}\vartheta (\cos^{2}\varphi -\sin^{2}\varphi ),$$ (72)

is rearranged in the two versions $h_{4}^{a}$ and $h_{4}^{b}$,

$$h_{4}^{a}=\sqrt{\frac{15}{16\pi }}\sin^{2}\vartheta \;(1-2\cdot \sin^{2}\varphi )=\sqrt{\frac{15}{16\pi }}\sin^{2}\vartheta -\sqrt{\frac{15}{4\pi }}\cdot \sin^{2}\vartheta \sin^{2}\varphi ,$$ (73)

$$\sin^{2}\vartheta \sin^{2}\varphi =-\sqrt{\frac{4\pi }{15}}h_{4}^{a}+\sqrt{\frac{1}{4}}\sin^{2}\vartheta$$ (74)

$$h_{4}^{b}=\sqrt{\frac{15}{16\pi }}\sin^{2}\vartheta \;(-1+2\cdot \cos^{2}\varphi )=-\sqrt{\frac{15}{16\pi }}\sin^{2}\vartheta +\sqrt{\frac{15}{4\pi }}\cdot \sin^{2}\vartheta \cos^{2}\varphi$$ (75)

$$\sin^{2}\vartheta \cos^{2}\varphi =\sqrt{\frac{4\pi }{15}}h_{4}^{b}+\sqrt{\frac{1}{4}}\sin^{2}\vartheta ,$$ (76)

with the use of cos^2^ *φ* + sin^2^ *φ* = 1. The second term in $h_{4}^{a}$ has the same form as the element $d_{1}^{2}$ and the second term in $h_{4}^{b}$ equals $d_{2}^{2}$. It is left to show, that the term sin^2^ *ϑ* can be expressed by the zero and the second-order real-valued spherical harmonic functions.

To see this the term $\sqrt{\frac{1}{4}}{\text{sin}}^{2}\vartheta$ will be rearranged,

$$\sqrt{\frac{1}{4}}\sin^{2}\vartheta =\sqrt{\frac{1}{4}}-\sqrt{\frac{1}{4}}\cos^{2}\vartheta =-\sqrt{\frac{1}{36}}\;(3\cos^{2}\vartheta -1)+\sqrt{\frac{1}{9}},$$ (77)

$$=-\sqrt{\frac{4\pi }{45}}h_{1}+\sqrt{\frac{4\pi }{9}}g_{0}$$ (78)

which is a linear combination of *h*_1_ and *g*_0_.

Together $d_{1}^{2}$ and $d_{2}^{2}$ can be expressed through the following spherical harmonics:

$$d_{1}^{2}=-\sqrt{\frac{4\pi }{15}}h_{4}-\sqrt{\frac{4\pi }{45}}h_{1}+\sqrt{\frac{4\pi }{9}}g_{0}$$

$$d_{2}^{2}=\sqrt{\frac{4\pi }{15}}h_{4}-\sqrt{\frac{4\pi }{45}}h_{1}+\sqrt{\frac{4\pi }{9}}g_{0}$$

### 5.4 The weight matrix of the original MFA

To see that Eq (20) corresponds to Eq (4) as derived by Franz et al. in 2004 [11] for the original MFA, a change of the basis of the vector space is applied. In [11] the coordinates of each flow vector ${\vec{p}}_{i}$ were given with respect to different basis vectors ${\vec{u}}_{i}$, ${\vec{v}}_{i}$, spanning the tangential plane on the sphere for viewing direction ${\vec{d}}_{i}$. In the following we derive the transformation matrix to transform the coordinates of a vector given in the standard Euclidean basis vectors ${\vec{e}}_{1}$, ${\vec{e}}_{2}$ and ${\vec{e}}_{3}$ into the basis defined by ${\left({\vec{u}}_{i},{\vec{v}}_{i},{\vec{d}}_{i}\right)}_{i=1,2,\ldots ,N}$. With *N* optical flow vectors we define a 3 × *N* dimensional vector space, which is the *N*-fold Cartesian product of the three basis vectors ${\vec{e}}_{1}$, ${\vec{e}}_{2}$ and ${\vec{e}}_{3}$.

All flow vectors ${\vec{p}}_{i}$ are represented now by a single stacked column vector $\vec{p}$,

$$\vec{p}=(\begin{array}{c} {\vec{p}}_{1}\\ {\vec{p}}_{2}\\ \vdots \\ {\vec{p}}_{N} \end{array}),$$ (79)

which has the dimension of 3 × *N*. In the same way the templates are written as

$${\vec{T}}_{A}=(\begin{array}{c} {\vec{T}}_{1,A}\\ {\vec{T}}_{2,A}\\ \vdots \\ {\vec{T}}_{N,A} \end{array}),$$ (80)

where the index *A* stands for one of the six standard templates. The six templates ${\vec{T}}_{A}$ are combined to matrix $\hat{T}$ with six columns and 3 × *n* rows:

$$\hat{T}=(\begin{array}{cccc} {\vec{T}}_{1,1} & {\vec{T}}_{1,2} & \cdots & {\vec{T}}_{1,6}\\ {\vec{T}}_{2,1} & {\vec{T}}_{2,2} & \cdots & {\vec{T}}_{2,6}\\ \vdots & \vdots & \ddots & \vdots \\ {\vec{T}}_{N,1} & {\vec{T}}_{N,2} & \cdots & {\vec{T}}_{N,6} \end{array}).$$ (81)

The responses of the six model neurons $\vec{a}$ in this notation are

$$\vec{a}={\hat{T}}^{T}\cdot \vec{p},$$ (82)

where ${\hat{T}}^{T}$ stands for the transpose of $\hat{T}$. Together with the coupling matrix one obtains for the self-motion components (see Eq (20)):

$${\vec{\theta }}^{\text{est}}={\hat{M}}^{-1}\cdot \vec{a}={\hat{M}}^{-1}\cdot {\hat{T}}^{T}\cdot \vec{p}.$$ (83)

The complete vector basis of the above templates and flow vectors is described by two indices *j* = 1,2,3 and *i* = 1, 2, …, *N* and has the form

$$B=({\vec{e}}_{1,1},{\vec{e}}_{2,1},{\vec{e}}_{3,1},{\vec{e}}_{1,2},{\vec{e}}_{2,2},{\vec{e}}_{3,2},\ldots ,{\vec{e}}_{1,N},{\vec{e}}_{2,N},{\vec{e}}_{3,N}),$$ (84)

where the ${\vec{e}}_{1,i}$, ${\vec{e}}_{2,i}$ and ${\vec{e}}_{3,i}$ are the same for each *i*. The basis vectors in the notation of Franz et al. [11] are

$$B^{\prime }=({\vec{u}}_{1},{\vec{v}}_{1},{\vec{d}}_{1},{\vec{u}}_{2},{\vec{v}}_{2},{\vec{d}}_{2},\ldots ,{\vec{u}}_{n},{\vec{v}}_{n},{\vec{d}}_{n}),$$ (85)

where the viewing directions ${\vec{d}}_{i}$ supplemented the local vector spaces to a three dimensional space.

The new basis vectors *B*′ can be expressed by the old *B*,

$${\vec{u}}_{i}=u_{1,i}{\vec{e}}_{1,i}+u_{2,i}{\vec{e}}_{2,i}+u_{3,i}{\vec{e}}_{3,i},$$

$${\vec{v}}_{i}=v_{1,i}{\vec{e}}_{1,i}+v_{2,i}{\vec{e}}_{2,i}+v_{3,i}{\vec{e}}_{3,i},$$

$${\vec{d}}_{i}=d_{1,i}{\vec{e}}_{1,i}+d_{2,i}{\vec{e}}_{2,i}+d_{3,i}{\vec{e}}_{3,i},$$

where *u*_1, *i*_ etc. is one component of ${\vec{u}}_{1}$. The transformation matrix $\hat{V}$ between the two bases with the dimension [3 × *N*] ⋅ [3 × *N*] is

$$\hat{V}=(\begin{array}{cccccccccc} u_{1,1} & u_{2,1} & u_{3,1} & 0 & 0 & 0 & \cdots & 0 & 0 & 0\\ v_{1,1} & v_{2,1} & v_{3,1} & 0 & 0 & 0 & \cdots & 0 & 0 & 0\\ d_{1,1} & d_{2,1} & d_{3,1} & 0 & 0 & 0 & \cdots & 0 & 0 & 0\\ 0 & 0 & 0 & u_{1,2} & u_{2,2} & u_{3,2} & \cdots & 0 & 0 & 0\\ 0 & 0 & 0 & v_{1,2} & v_{2,2} & v_{3,2} & \cdots & 0 & 0 & 0\\ 0 & 0 & 0 & d_{1,2} & d_{2,2} & d_{3,2} & \cdots & 0 & 0 & 0\\ \vdots & \vdots & \vdots & \vdots & \vdots & \vdots & \ddots & \vdots & \vdots & \vdots \\ 0 & 0 & 0 & 0 & 0 & 0 & \cdots & 0 & 0 & 0\\ 0 & 0 & 0 & 0 & 0 & 0 & \cdots & u_{1,N} & u_{2,N} & u_{3,N}\\ 0 & 0 & 0 & 0 & 0 & 0 & \cdots & v_{1,N} & v_{2,N} & v_{3,N}\\ 0 & 0 & 0 & 0 & 0 & 0 & \cdots & d_{1,N} & d_{2,N} & d_{3,N} \end{array}).$$

The matrix $\hat{V}$ transforms the coordinates of a vector given in basis *B*, Eq (84), into basis *B*′, Eq (85). Eq (83) becomes

$$\begin{array}{ccc} {\vec{\theta }}^{\text{est}} & = & {\hat{M}}^{-1}\cdot {\hat{T}}^{T}\cdot \vec{p}={\left({\hat{T}}^{T}\cdot \hat{T}\right)}^{-1}\cdot {\hat{T}}^{T}\cdot \vec{p}\\ & = & {\left({\hat{T}}^{T}\cdot {\hat{V}}^{T}\cdot \hat{V}\cdot \hat{T}\right)}^{-1}\cdot {\hat{T}}^{T}\cdot {\hat{V}}^{T}\cdot \hat{V}\cdot \vec{p}\\ & = & {\left({\left(\hat{V}\cdot \hat{T}\right)}^{T}\cdot \hat{V}\cdot \hat{T}\right)}^{-1}\cdot {\left(\hat{V}\cdot \hat{T}\right)}^{T}\cdot \hat{V}\cdot \vec{p}. \end{array}$$

The term $\hat{V}\cdot \vec{p}$ is equivalent to $\vec{x}$ in Eq (4) from the original MFA of Franz et al. [11]. The term $\vec{x}$ combines all optic flow components of the local two dimensional vector spaces as spanned by the two local vectors $\vec{u}$ and $\vec{v}$. The third component is zero because $\vec{p}$ and $\vec{d}$ are orthogonal.

To see that $\hat{V}\cdot \hat{T}$ is $F={\left(\begin{array}{c} -{\bar{\mu }}_{1}{\vec{u}}_{1}\\ {\vec{u}}_{1}\times {\vec{d}}_{1} \end{array},\begin{array}{c} -{\bar{\mu }}_{1}{\vec{v}}_{1}\\ {\vec{v}}_{1}\times {\vec{d}}_{1} \end{array},\ldots \right)}^{T}$ the first row of $\hat{V}\cdot \hat{T}$ is scrutinized. The six components are

$${\vec{u}}_{1}\cdot {\vec{T}}_{1,1},\;{\vec{u}}_{1}\cdot {\vec{T}}_{1,2},\;{\vec{u}}_{1}\cdot {\vec{T}}_{1,3},\;{\vec{u}}_{1}\cdot {\vec{T}}_{1,4},\;{\vec{u}}_{1}\cdot {\vec{T}}_{1,5},\;{\vec{u}}_{1}\cdot {\vec{T}}_{1,6}.$$

The first three components are (*j* = 1, 2, 3)

$$-\mu_{1}\cdot {\vec{u}}_{1}\cdot ({\vec{d}}_{1}\times {\vec{e}}_{j}\times {\vec{d}}_{1})=-\mu_{1}\cdot {\vec{u}}_{1}\cdot ({\vec{e}}_{j}-({\vec{e}}_{j}\cdot {\vec{d}}_{1}){\vec{d}}_{1})=-\mu_{1}\cdot {\vec{u}}_{j,1},$$

and the second three components are

$$-{\vec{u}}_{1}\cdot ({\vec{e}}_{j}\times {\vec{d}}_{1})={\vec{e}}_{j}\cdot ({\vec{u}}_{1}\times {\vec{d}}_{1})={\left({\vec{u}}_{1}\times {\vec{d}}_{1}\right)}_{j}.$$
